# Interaction of HS1BP3 with cortactin modulates TKS5 localisation, cell secretion and cancer malignancy

**DOI:** 10.1002/1878-0261.70248

**Published:** 2026-04-10

**Authors:** Arja Arnesen Løchen, Kristiane Søreng, Chiara Veroni, Laura Trachsel‐Moncho, Nagham Asp, Robin Gaupset, Lars Gustav Lyckander, Helene Knævelsrud, Lars Eftang, Anne Simonsen

**Affiliations:** ^1^ Department of Molecular Medicine, Institute for Basic Medical Sciences, Faculty of Medicine University of Oslo Norway; ^2^ Centre for Cancer Cell Reprogramming, Institute of Clinical Medicine, Faculty of Medicine University of Oslo Norway; ^3^ Department of Molecular Cell Biology, Institute for Cancer Research Oslo University Hospital Norway; ^4^ Department of Microbiology and Infection Control Akershus University Hospital Norway; ^5^ Department of Digestive Surgery Akershus University Hospital Norway; ^6^ Department of Pathology Akershus University Hospital Norway

**Keywords:** cortactin, endosomes, HS1BP3, invasion, proliferation, TKS5

## Abstract

The HCLS1‐Binding Protein 3 (HS1BP3) interacts with the SH3 domain of cortactin (CTTN), a protein that contributes to a malignant phenotype in cancers. Here, we demonstrate that high expression of HS1BP3 is associated with reduced survival for gastric adenocarcinoma and triple negative breast carcinoma patients and that HS1BP3 is specifically upregulated in these cancers. We mapped the HS1BP3‐cortactin interaction site to the third proline‐rich region (PRR3.1) of HS1BP3 and show that this interaction is important for cancer cell proliferation, extracellular matrix degradation and secretion. HS1BP3 expression was found to correlate with expression of the invadopodia scaffold protein TKS5 and we show that the localisation of TKS5 inside multivesicular endosomes is increased in cells expressing an HS1BP3 PRR3.1 mutant. Overall, our results highlight the importance of the direct interaction between HS1BP3 and cortactin in cancer development by regulating cell proliferation, secretion and invasion, which may provide an explanation for the negative correlation between HS1BP3 levels and the survival of gastric adenocarcinoma and triple negative breast cancer patients.

## Graphical abstract

Here, we demonstrate that HS1BP3 interacts with Cortactin through a proline‐rich region (PRR3.1) and show that this interaction, and HS1BP3 itself, promote cancer cell proliferation and invasion. Inhibition of this interaction leads to build‐up of TKS5 in multivesicular endosomes and altered secretion of CD63 and CD9, providing an explanation for the inverse correlation between HS1BP3 levels and cancer patient survival.

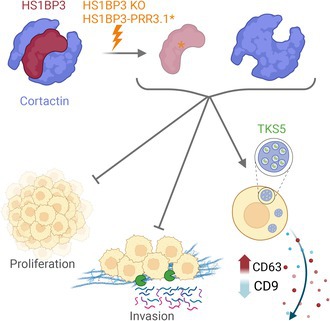

**Abbreviations**
CTTN: cortactin
F‐actin: filamentous actin
HR: hazard ratio
HS1BP3: HCLS1‐binding protein 3
KM: Kaplan Meier
LAMP1: lysosome associated membrane glycoprotein 1
LC3: microtubule‐associated protein light‐chain 3 protein
MVB: multivesicular bodies
PRR: proline rich region
SH3: Src homology domain 3
TNBC: triple negative breast cancer

## 1 Introduction

The aggressiveness of cancers is associated with their cell proliferation rate and ability to migrate and degrade extracellular matrix to allow metastasis [1]. These attributes are tightly regulated by the interplay between complex molecular pathways, including endosomal trafficking [2] and autophagy [3], as well as several factors involved in cytoskeletal remodelling [4].

Endocytosis involves the internalisation of extracellular and plasma membrane localised components into endocytic vesicles that are further sorted for either lysosomal degradation, recycling to the plasma membrane or secretion [2]. Several proteins involved in endosomal trafficking have been implicated in tumour metastasis, often associated with the regulation of cellular motility and invasion through the basement layer [2]. Endocytosis also crosslinks with the processes of autophagy that involve uptake of cytoplasmic material into double‐membraned autophagosome vesicles that fuse with the lysosome for content degradation [5].

We have previously identified and characterised the HCLS1‐binding protein 3 (HS1BP3) as a negative regulator of starvation‐induced bulk autophagy and hypoxia‐induced autophagy of mitochondria [6, 7, 8]. HS1BP3 localises on recycling endosomes and was found to interact directly with the Src homology 3 domain (SH3) domain of the Src Substrate cortactin (cortactin) [7]. Cortactin regulates the actin cytoskeleton through interaction with the actin‐related protein 2/3 complex (Arp2/3) and filamentous(F)‐actin [9, 10] and contains an SH3 domain that interacts with several proteins involved in actin cytoskeleton reorganisation [11].

Cortactin is important for cancer cell metastasis through its regulation of actin‐cytoskeleton dynamics, permitting cancer cell migration, invadopodia formation and matrix degradation [11, 12, 13, 14]. Additionally, cortactin regulates endosome trafficking [15, 16], the secretion of multivesicular endosome‐derived exosomes important for migration [17] and extracellular matrix invasion [18]. Cortactin also interacts with the Src substrate and invadopodia scaffold protein TKS5 at the initial stages of invadopodia/podosome formation [14, 19].

HS1BP3 has recently been associated with regulation of hepatocellular carcinoma progression [20]. We wanted to further examine the role of HS1BP3 in tumorigenesis and investigate whether it regulates cancer development via its interaction with cortactin and/or by regulation of autophagy or the endocytic pathway. Here, we demonstrate that high expression of HS1BP3 may be predictive of poor prognosis in gastric adenocarcinoma and triple negative breast cancer (TNBC). We identify a proline‐rich region (PRR) in HS1BP3 important for its direct interaction with cortactin and show that cancer cell proliferation and matrix substrate invasion, as well as the localisation and stability of TKS5 and amount of cell secretion, depend on this interaction.

## 2 Materials and methods

### 2.1 Reagents and tools

A detailed list of all reagents and tools used in this study can be found in the Supporting Information (Table S1).

### 2.2 Methods and protocols

#### 2.2.1 KM plot analysis

Kaplan–Meier plot analysis and Cox proportional hazards regression on overall survival or relapse‐free survival are based on mRNA gene chip data from breast, ovarian, lung, gastric, colon, AML, and myeloma cancer patients and were performed using the KM‐plotter database and interface www.kmplot.com [21, 22]. Comparison groups were split according to high (third quartile) and low (first quartile) levels of the gene of interest by trichotomization. For the effect of HS1BP3 expression, indicated Hazard ratios with 95% confidence intervals and *P*‐values were plotted on a forest plot for comparison on the effect of HS1BP3 in different cancers.

#### 2.2.2 Gene expression set analysis

To study gene expression data of healthy and cancerous tissues, Xena [23] was used to filter GTex Portal and TCGA data for the gene(s) and tissue(s) of interest.

#### 2.2.3 AlphaFold model

The interaction between the full‐length HS1BP3 (Q53T59) and the SH3 fragment of cortactin (Q14247: amino acids 492–550) was modelled in AlphaFold Server (AlphaFold V3) https://alphafoldserver.com/ [24]. All five ranked models were investigated using Pymol version 3.1.3 (Schrödinger, LLC) where all models had similar interactions and one is shown.

The InterfaceResidues script https://www.pymolwiki.org/index.php/InterfaceResidues was used to illustrate interacting residues in cloud view and the Konstantin Korotkov code was used to show AlphaFold pLDDT colour scheme https://github.com/sokrypton/ColabFold.

#### 2.2.4 Cell culture

AGS (CRL‐1739; RRID: CVCL_0139), NCI‐N87 (CRL‐5822; RRID: CVCL_1603) and MDA‐MB‐231 (HTB‐26; RRID: CVCL_0062) were purchased from ATCC, and MKN‐74 (JCRB0255; RRID: CVCL_2791) was purchased from JC3B cell bank. AGS were cultured in Ham's F‐12 K (Kaighn's) Medium (Gibco, 21127030; Paisley, UK) supplemented with 10% Fetal Bovine Serum (FBS; Sigma Aldrich, #F7524; Steinheim, Germany) and 1% penicillin–Streptomycin mix (Thermo‐Fisher Scientific, #15140122; Grand Island, NY, USA), while MDA‐MB‐231, MKN‐74 and NCI‐N87 cells were maintained in RPMI 1640 Medium (Gibco, 61870–044) in 10% FBS and 1% PS. All cells were cultured in a humidified incubator at 37 °C with 5% CO_2_. MKN‐74 and MDA‐MB‐231 cells with stable overexpression of HS1BP3 were kept in the indicated medium with appropriate resistance antibiotics. To starve the cells in nutrient‐deplete medium, they were incubated in Earle's Balanced Salt Solution (EBSS; Gibco, 24010043). MDA‐MB‐231 WT cells and MDA‐MB‐231 cells stably overexpressing TKS5‐GFP were a kind gift from C. Raiborg [25]. All cells used in this study have been testing negative for mycoplasma and authenticated with short tandem repeat analysis in the past three years of use.

#### 2.2.5 Generation of MKN‐74 HS1BP3 KO

To generate MKN‐74 HS1BP3 KO cells, CRISPR/Cas9 KO plasmids (px459) [26] were digested with BbSI and forward and reverse guide RNAs 1 (fw 5′CACCGCGCCGTCATGCAGTCCCCGG3′, rv 5′AAAC CCGGGGACTGCATGACGGCG3′) and 2 (fw 5′CACCgCCCCAGCACCAGGAGGTACG3′, rv 5′AAAC CGTACCTCCTGGTGCTGGGGc3′) were annealed respectively, BbSI‐digested and ligated into the digested px459 plasmid. MKN‐74 cells were transfected with the CRISPR/Cas9 KO plasmids using Lipofectamine 2000 (Invitrogen, 11 668 019; Carlsbad, CA, USA) and subsequently selected for positive clones for a maximum of 48 h with 2 μg·mL^−1^ puromycin (Sigma‐Aldrich, P7255‐25MG; Saint Louis, MA, USA).

#### 2.2.6 Plasmids

All plasmids were made using Gibson Assembly master mix (NEB, E2611L), Gateway™ LR Clonase™ reaction (Invitrogen, 11 791–100) or the QuikChange II site‐directed mutagenesis kit (Agilent, 210 513; Cedar Creek, TX, USA) using primers as indicated in Table S1.

#### 2.2.7 Generation of stably overexpressing cells

MKN‐74 WT and HS1BP3 KO cells and MDA‐MB‐231 cells with stable overexpression were made by co‐transfecting indicated expression plasmids with psPAX2 and pCMV‐VSVG [27] at a 1 : 1 : 1 ratio using Xtreme‐GENE DNA 9 Transfection Reagent (Roche, XTG9‐RO) into HEK293FT cells to produce lentiviral particles that were transduced into target cells. Positive cells were selected with 1 μg·mL^−1^ puromycin (Sigma‐Aldrich, P7255‐25MG), 0.5 mg·mL^−1^ Geneticin (Thermo Fisher Scientific, 11–811‐031; Waltham, MA, USA) or 100 μg·mL^−1^ zeocin (ThermoFisher Scientific, R25001) depending on the antibiotic resistance of the transduced expression plasmid.

#### 2.2.8 Transient overexpression of EGFP and TKS5‐EGFP

For transient transfection of EGFP (pEGFP‐N1) and TKS5‐EGFP (pEGFP‐TKS5‐N1), 8.0 × 10^5^ MKN‐74 cells were seeded into 10‐cm dishes and left for 48 h before transfection of the indicated plasmids at equimolar concentrations of each gene using lipofectamine 2000 (Invitrogen, 11 668 019).

#### 2.2.9 siRNA knockdown

Cells were incubated with Dharmacon oligonucleotides targeting each gene at 20 nm, in combination with RNAiMAX (Invitrogen, 13 778 150) through reverse transfection for 72 h. The siRNA nucleotides used were as follows: siCTRL (5′UGGUUUACAUGUCGACUAA3′, #D‐001810‐01–20), siHS1BP3 (5′UGAAGAGGCUUUCGACUUU3′, #J‐013029‐10‐0020).

#### 2.2.10 *In vitro* protein production and purification

GST‐ and MBP‐tagged proteins were expressed in SoluBL21 *E. coli* cells. GST‐bound proteins were purified by lysing the *E. coli* cells with GST‐lysis buffer (50 mm Tris–HCl (pH 8; Sigma‐Aldrich, T1378‐1KG), 250 mm NaCl (Sigma‐Aldrich, 71 376), 1 mm EDTA (Roche, 10 708 984 001, Basel, Switzerland), 1× complete EDTA‐free protease inhibitor cocktail (Roche, 5 056 489 001)) and isolating the proteins with Glutathione Sepharose^®^ 4B beads (Sigma Aldrich, GE17‐0756‐01; Uppsala, Sweden). MBP‐tagged proteins were purified by lysing the *E. coli* cells in MBP‐lysis buffer (20 mm Tris–HCl pH 7.4, 200 mm NaCl, 1 mm EDTA, 1 mm DTT, 1× complete EDTA‐free protease inhibitor cocktail) and isolating the proteins with amylose‐resin beads (NEB, E8021L; Ipswich, MA, USA), followed by elution with 10 mm maltose on Poly‐Prep^®^ Chromatography Columns (Biorad, 7 311 550; Richmond, CA, USA) at 4 °C. For *in vitro* interaction assays, equal concentrations of GST‐ and MBP‐tagged proteins were incubated together for 2 h at 4 °C and washed six times with GST‐buffer before heat inactivation and denaturation at 95 °C in Laemmli Sample Buffer (Bio‐Rad, 1 610 747; Hercules, CA, USA) supplemented with DTT (Roche, 10 708 984 001) followed by immunoblotting. The same procedure was done for cell lysate incubation with GST‐tagged proteins; here, 450 μg protein lysate (as measured using Pierce™ BCA™ Protein assay kit (Thermo Scientific; 23235, Rockford, IL, USA)) was added.

#### 2.2.11 Cell lysis and immunoprecipitation

Cells were lysed in RIPA buffer (50 mm Tris–HCl (pH 7.5; Sigma‐Aldrich, T1378‐1KG), 150 mm NaCl (Sigma‐Aldrich, #71376), 1 mm EDTA (Sigma‐Aldrich, #60‐00‐4), 0.05% Nonidet P‐40 Substitute (Roche, 11 754 599 001), 0.25% Triton X‐100 (Sigma‐Aldrich, #9002‐93‐1)) and centrifuged at 15 000 × ***g*** for 10 min. The protein concentration of the soluble protein fraction was measured using the Pierce™ BCA™ Protein assay kit (Thermo Scientific, 23235). For GFP‐trap assays, at least 450 μg protein lysate or the whole amount of lysate was added to 15 μL homogenised and precleared ChromoTek GFP‐Trap^®^ Agarose beads (Proteintech, Gta‐20;Chro Planegg‐Martinsried, Germany) for 2.5 h before washing the beads four times in RIPA buffer before heat inactivation and denaturation at 95 °C in Laemmli Sample Buffer (Bio‐Rad, 1 610 747) supplemented with DTT (Roche, 10 708 984 001) before immunoblotting.

#### 2.2.12 Patient sample storage and handling

Biopsies were obtained from 28 patients diagnosed with gastric adenocarcinoma at Akershus University Hospital, Norway, in the years 2020–2022. On admission for surgery, each participant gave a written, informed consent to participate in the study. Procedures conducted included complete gastrectomy or partial gastrectomy depending on the tumour location in the stomach. The biopsies were taken within 10 min after removal of the surgical specimen. From each patient, two tumour samples as well as two control samples from healthy gastric tissue adjacent to the tumour were obtained. One set of tumour and control samples were directly placed in Allprotect tissue reagent (Qiagen, 76.405; Hilden, Germany) for long‐term storage at −20 °C, and the other set was directly stored in 10% formalin solution for immunohistochemistry analysis and placed at 4 °C. After 24 h, the formalin was diluted to 1% for prolonged storage at 4 °C. RNA extraction of Allprotect samples is described below.

#### 2.2.13 Immunohistochemistry

To perform immunohistochemistry (IHC) on gastric samples from gastric cancer patients, formalin‐fixed tissue samples were paraffin‐embedded with the gastric mucosal lining stretched out flat and sectioned to 3 μm sections. The sections were deparaffinised using Dako PT‐link system with Lab Vision™ PT Module and a high pH buffer (EnVision™ FLEX Target Retrieval Solution) and the IHC was done using the Dako EnVision Flex+System/EnVision+System‐HRP (DAB) (K4007) with a primary HS1BP3 antibody. All antibodies were diluted in Dako Antibody Diluent (#S0809) and a secondary negative antibody control (diluent‐only) was prepared to ensure specific staining. The sections were then haematoxylin‐stained to view cell nuclei before section dehydration and mounting in Cytoseal XYL. The sections were then imaged high‐throughput at 40× brightfield with an Olympus VS200 Slide Scanner and analysed for levels of HS1BP3 staining using QuPath with the ‘positive cell detection mode’ in colour‐deconvoluted channels [28]. At least 14 000 cells were segmented and quantified per sample.

#### 2.2.14 RNA isolation and qPCR

Cells were trypsinised and washed in PBS before RNA extraction using the RNeasy plus mini kit (Qiagen, 74 136) according to the manufacturer's instructions. To extract RNA from the Allprotect‐stored biopsies, the AllPrep DNA/RNA/Protein Mini Kit (Qiagen, 80 004) was used according to the manufacturer protocol with TissueRuptor I (Qiagen) homogenisation of the tissue and additional on‐column DNA digestion with the RNase‐Free DNase Set (Qiagen, 79 254). The RNA was reverse transcribed using the SuperScript™ III Reverse Transcriptase (Thermo fisher scientific, 18 080 085) and qPCR was performed using KAPA SYBR^®^ FAST qPCR Kit (KAPA BIOSYSTEMS, KK4601; Cape Town, South Africa) in a CFx96 real‐time PCR system (Bio‐Rad) with Qiagen QuantiTect primers (Qiagen, 249 900). Transcript levels of genes of interest were normalised to SDHA and fold change quantifications were performed using the 2^−ΔΔCt^ method.

#### 2.2.15 Protein extraction and immunoblotting

Cells were lysed in RIPA buffer (either: 50 mm Tris–HCl (pH 7.5; Sigma‐Aldrich, T1378‐1KG), 150 mm NaCl (Sigma‐Aldrich, #71376), 1 mm EDTA (Sigma‐Aldrich, #60‐00‐4), 0.05% Nonidet P‐40 Substitute (Roche, 11 754 599 001), 0.25% Triton X‐100 (Sigma‐Aldrich, #9002‐93‐1); or: 20 mm Tris–HCl (pH 7.4), 150 mm NaCl, 5 mm EDTA and 1% Triton X‐100) and protein concentration was measured using the Pierce™ BCA™ Protein assay kit (VWR, 786–0000) and denatured at 95 °C in Laemmli Sample Buffer (Bio‐Rad, 1 610 747) supplemented with DTT (Roche, 10 708 984 001). At least 20 μg of protein was loaded on 4–20% gradient acrylamide gel (BioRad, 5 678 094) and transferred to a PVDF membrane. For Ponceau S staining, the membrane was dried and rewetted with 100% methanol before Ponceau S staining followed by membrane drying and imaging in the ChemiDoc MP (BioRad) imaging system. The membranes were blocked in either 5% nonfat milk (VWR, A0830.0500) or 1% casein (Sigma Aldrich, C7078‐500G) in 1× PBS for a minimum of 45 min before primary antibody incubation in 5% bovine serum albumin (Sigma Aldrich, A1470‐100G) in 1× PBS overnight at 4 °C. The membranes were washed and incubated with secondary antibody diluted in 1× PBS for a minimum of 45 min at room temperature before washing and imaging. For infrared fluorescent signal, the Odyssey^®^ CLx (LI‐COR^®^) and ChemiDoc MP were used to image the membranes. For chemiluminescent signal, the SuperSignal West Pico PLUS Chemiluminescent Substrate (Thermo Fisher, 34 577) was used according to manufacturer instructions and the signal detected in the ChemiDoc MP. Band quantifications were done in Image Studio™Lite and Fiji [29].

#### 2.2.16 Immunofluorescence

For immunofluorescence, cells in complete growth medium were fixed by directly adding 2× 4% PFA‐PHEM buffer and incubating the cells for 15 min at 37 °C with 1 : 10 000 Hoechst 33342 (Sigma Aldrich, 1 μg·mL^−1^). Cells were then washed 3× in PBS and incubated with primary antibody diluted in 0.05% saponin in PBS‐BSA 3% for 1–2 h. After three washes in PBS, the cells were incubated with the corresponding secondary antibodies diluted in PBS for 40 min‐1 h and finally washed 3× in PBS. If cells were grown on coverslips, the coverslips were mounted in ProLong™ Diamond Antifade Mountant (Invitrogen, P36961). If grown in 8‐well chambers, the cells were kept in PBS with 0.02% NaAzide (VWR, AA14314‐36) at 4 °C. Image analysis was carried out using CellProfiler software (v4.2 and 4.2.5) [30]. The images were separated into individual channels and in some experiments enhanced to improve the visibility of key features. Nuclei, cells and puncta were detected by applying a manual threshold to segment the areas of interest. Colocalisation was assessed by relating the structures. Pixel intensity plots were generated with Fiji ImageJ. A line was drawn on the region of interest and pixel intensity values for each channel were extracted and plotted along the length of the line.

For MKN‐74 cells, the glass coverslip/8‐well chamber bottom was coated with poly‐d‐lysine (Merck, P6403; Darmstadt, Germany) for a minimum of 30 min at 37 °C and washed 1× with PBS before cell seeding to ensure attachment.

#### 2.2.17 Wound healing assay

Cells were seeded to confluency in 96‐well ImageLock™ plates (Essen Bioscience, 4379) and left overnight. To make wounds, the Incucyte^®^ 96‐well WoundMaker tool (Essen Bioscience, Hertfordshire, UK) was used and the medium changed before the plate was left for up to 80 h in the IncuCyte^®^ S3 Live Cell microscope (Sartorius) for imaging every 10 min or 30 min. For experiments including Mitomycin C treatment, 6 μg·mL^−1^ Mitomycin or no treatment was added at the start of imaging. Quantification of relative wound density (compared to time 0 h) was done using the incucyte^®^ software S3 (Sartorius) by measuring the ‘spatial cell density’ in the wound area relative to the spatial cell density outside of the wound area at every time point.

#### 2.2.18 Cell viability assays

For the MTT assay, cells were seeded to 70% confluence in 96‐well plates and left overnight before adding MTT viability reagent (Thiazolyl Blue Tetrazolium Bromide, Merck, M2128) to a working concentration of 0.5 mg·mL^−1^ for four hours at 37 °C in a humidified chamber. The liquid was then removed, and the resulting formazan crystals were dissolved in DMSO before measuring absorbance at 570 nm.

To measure trypan blue incorporation, the same number of cells of each cell type were seeded in T‐75 tissue culture plates and cultured for three days, followed by cell trypsinisation and counting with Trypan reagent in Invitrogen™ Countess™ Cell Counting Chamber Slides (Invitrogen, 10 399 053). The total number of cells with and without Trypan Blue incorporation was counted.

#### 2.2.19 Proliferation assays

Cells were seeded to 2–10% confluency in 96‐well plates and left for 51 h in IncuCyte^®^ S3 Live Cell microscope (Sartorius) with imaging every 30 min or 1.5 h. Confluence was quantified in the incucyte^®^ software S3 (Sartorius), and data presented as relative confluence compared to time 0 using the mean of three replicate wells from three independent experiments. For experiments including Mitomycin C treatment, 6 μg·mL^−1^ Mitomycin or no treatment was added at the start of imaging.

#### 2.2.20 Gelatin degradation assay

To prepare gelatin‐coated coverslips, 12 mm diameter (VWR, 631‐0666; Marienfield, Germany) coverslips were sterilised by laying flat on parafilm in laminar flow cell hood with UV light treatment for 60 min. Then, while continuing to work in the cell hood, 30 μL preheated (37 °C) 0.2 mg·mL^−1^ Gelatin Oregon Green 488‐conjugate (Invitrogen, G‐13186) in 2% sucrose in PBS was dropped on clean parafilm and the sterilised coverslips left on top for 20 min at room temperature in the dark. 20 μL of 0.5% glutaraldehyde in PBS was left on a clean sheet of parafilm and the gelatin‐coated coverslips moved on top gelatin‐side down. The coated coverslips were fixed for a minimum of 40 min followed by 3x wash in PBS and kept in 1% penicillin–streptividin in PBS at 4 degrees until use. Before use, the coverslips were incubated in complete cell culture medium for at least 60 min before seeding cells on top for the indicated time and subsequently fixed in 4% paraformaldehyde in PHEM for 15 min at 37 °C. The cells were subsequently permeabilised in 0.05% saponin and stained in 1 : 200 solution of Phalloidin‐633 (Invitrogen, A22284) and 1 : 10 000 Hoechst 33342 (Sigma Aldrich, 1 μg·mL^−1^) in 0.05% saponin in PBS for 40 min before 3× wash in PBS and mounting in ProLong™ Diamond Antifade Mountant (Invitrogen, P36961). The number of cells covering degradative areas in the gelatin (completely black spots) were counted and compared relative to the total number of cells observed.

#### 2.2.21 Extracellular vesicle enrichment

Extracellular vesicle enrichment using 10 K spin‐column filtration was done as described previously [25]. Briefly, 6 × 10^6^ cells were seeded in 150‐mm dishes and incubated for 48 h before washing the dishes 2× in 12 mL serum‐free RPMI (Gibco, 61 870–044) followed by incubation for 44 h in 14 mL serum‐free RPMI. Then, the conditioned media was centrifuged at 300 × ***g*** at 4 °C for 10 min, at 1000 × ***g*** at 4 °C for 10 min, and at 10 000 × ***g*** at 4 °C for 30 min. This was followed by concentration of the extracellular vesicle fraction to about 300 μL using an AmiconUltra‐15 centrifugal filter unit (UFC901024) at 4000 × ***g*** for 8 min. The cells were trypsinised and counted, followed by western blot analysis of 10% of the whole cell lysate and 50% of the concentrated media.

#### 2.2.22 Statistical analysis

Statistical analysis and preparation of figures, except for the KM‐analysis, were performed using Graphpad Prism with tests as indicated in the figure legends.

#### 2.2.23 Ethical considerations

This project has been under the necessary ethical considerations and the collection, storage and use of gastric tissue from gastric adenocarcinoma patients was pre‐approved by the Norwegian Regional committee for medical and health research ethics (reference #166245). The study methodologies conformed to the standards set by the Declaration of Helsinki.

## 3 Results

### 3.1 High expression of *HS1BP3* is associated with reduced survival of gastric adenocarcinoma and TNBC patients

**Fig. 1:**
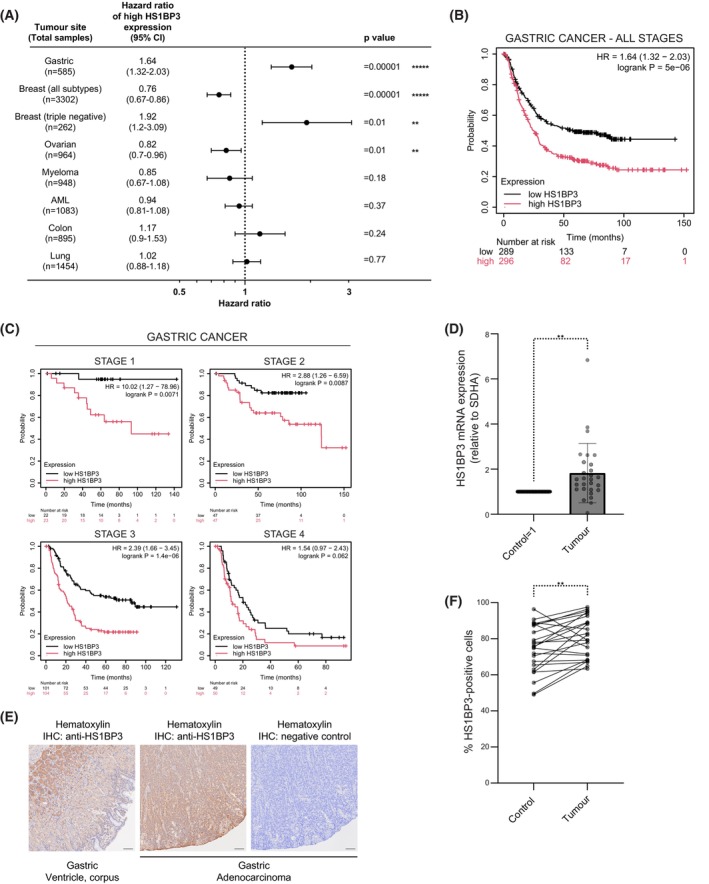
*HS1BP3* levels negatively correlate with survival of gastric cancer patients. (A) Forest plot showing the hazard ratio plotted on a Log_10_ scale with 95% confidence interval (CI) of high (T3 = third highest expressing tertile) mRNA expression of *HS1BP3* compared to low (T1 = third lowest expressing tertile) *HS1BP3* expressing patients in eight cancer types. (B, C) KM plots showing the probability of survival in patients with gastric cancer according to high (T3 = third highest expressing tertile) and low (T1 = third lowest expressing tertile) mRNA expression of *HS1BP3* in tumour at time of detection at all cancer stages (B) and according to cancer stage (C). (A–C) The hazard ratio, CI and *P*‐values were calculated using the Cox proportional hazards regression tool in the Kaplan Meier (KM) plotter database. (D–F) Healthy control and tumour gastric tissue from 28 gastric adenocarcinoma patients were pairwise analysed for *HS1BP3* mRNA expression using quantitative PCR (D) or for HS1BP3 protein expression using IHC + haematoxylin stains (E, F). (E) shows representative parts of the imaging of the anti‐HS1BP3 IHC samples from one patient together with a negative secondary IHC stain control of the tumour sample, the scale bars are 100 μm. (D) shows the mean and standard deviation relative to the control sample (1) (F) show the pair‐wise percentage values between the control and tumour sample per patient. (D–F) *P* values were determined using paired t‐test. (A, D, F) **P < 0.01; *****P < 0.00001.

To investigate a possible role of HS1BP3 in tumorigenesis, we first used the Kaplan–Meier plot analysis tool to address whether *HS1BP3* gene expression levels may predict the survival of cancer patients [21, 22]. Interestingly, high *HS1BP3* expression was a strong predictor of poor survival of gastric cancer patients (Fig. 1A,B: hazard ratio HR = 1.64) and patients with the TNBC subtype (Fig. 1A, Fig. S1A,B; HR = 1.92), an aggressive subtype that is depleted of oestrogen and progesterone receptors and HER2 growth factor [31]. *HS1BP3* expression in tumour tissue was also significantly but weakly predictive of improved survival in all subtypes of breast (HR = 0.76) and ovarian (HR = 0.82) cancer patients (Fig. 1A, Fig. S1A,C). There were no significant associations between *HS1BP3* expression levels and cancer patient survival in other cancer types. This suggests that HS1BP3 may act as a tumour‐promoter in TNBC and gastric cancer specifically.

When analysing the correlation between *HS1BP3* levels and patient survival with respect to gastric cancer development stage, we found that a high HS1BP3 expression level was a significant and strong predictor of poor outcomes among gastric cancer patients at Stages 1–3 (HR = 10.02, 2.88, 2.39 respectively) but not at Stage 4 (HR = 1.57, *P* > 0.05), indicating that *HS1BP3* has an important role in the early phase of cancer development (Fig. 1C). Using the GTex portal to analyse *HS1BP3* mRNA expression levels in different tissues, we found *HS1BP3* to be highly expressed at the gastro‐oesophageal junction and in stomach tissues (Fig. S1D). Moreover, comparison with the TCGA database demonstrated that expression of *HS1BP3* is significantly higher in stomach adenocarcinoma and breast carcinoma than in healthy tissue (Fig. S1E,F).

We next collected healthy and tumour gastric tissue from 28 gastric adenocarcinoma patients undergoing gastrectomy surgery, followed by pair‐wise comparison of HS1BP3 mRNA levels and the amount of HS1BP3 staining in immunohistochemistry (IHC) sections for each patient (Fig. 1D–F). Overall, these data showed a significant increase in *HS1BP3* expression levels in the tumour compared to the healthy control tissue, demonstrating that *HS1BP3* is specifically upregulated in gastric adenocarcinoma tissue.

### 3.2 HS1BP3 is important for the proliferation of gastric adenocarcinoma cells

To examine the role of HS1BP3 in the early stages of gastric cancer, we acquired three immortalised gastric carcinoma cell lines: AGS, NCI‐N87 and MKN‐74. While the AGS cell line is derived from a primary gastric adenocarcinoma site, the NCI‐N87 and MKN‐74 cell lines are sampled from liver metastases of gastric adenocarcinomal origin.

**Fig. 2:**
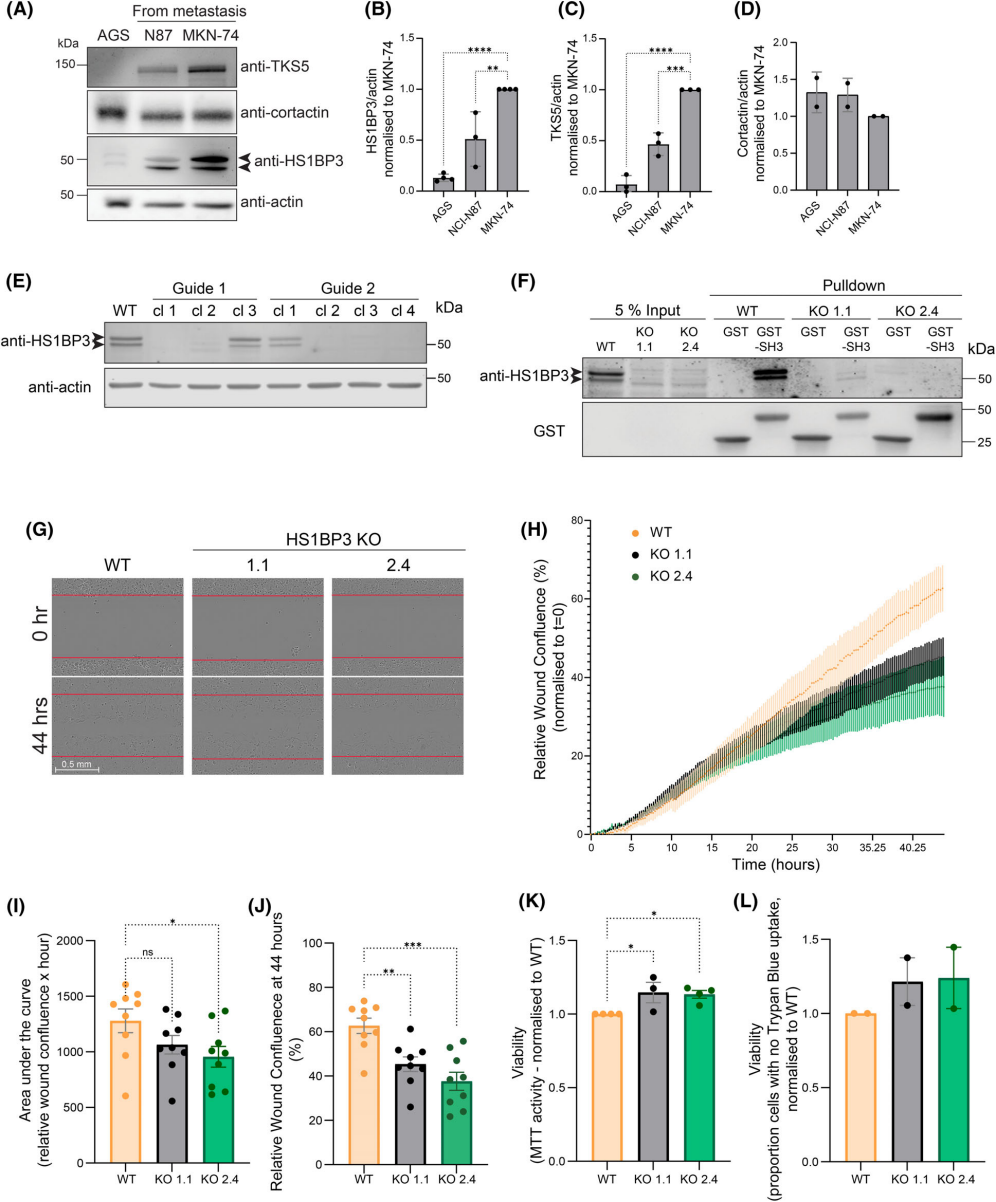
HS1BP3 Promotes migration of a gastric cancer cell line. (A) Immunoblotting and quantification of protein levels of TKS5, cortactin, and HS1BP3 in three gastric cancer cell lines (AGS, N87 and MKN‐74). (B–D) Quantification of (A) shows the mean level of the indicated protein relative to Actin levels and normalised to levels in MKN‐74. *N* = 2–4 (as indicated by the number of points on the plot). (E) Lysates of MKN‐74 wild‐type (WT) cells and single MKN‐74 clones subjected to CRISPR/Cas9 knockout (KO) of HS1BP3 using two different gRNA (guide 1 and guide 2) were subjected to immunoblotting against HS1BP3. Actin was used as a loading control, *n* = 1. (F) GST‐SH3 (SH3 domain of cortactin) pulldown of HS1BP3 from MKN‐74 lysates of WT and two single HS1BP3 KO clones (clone 1.1 and 2.4). *n* = 1. (G, H) MKN‐74 WT and HS1BP3 KO clones (1.1 and 2.4) were seeded confluently in 96‐well plates and left overnight before creating wounds and imaged by live cell microscopy every ten minutes over 44 h to quantify the filling of the wound. *n* = 3. (G) Representative images of cells at 0 h or 44 h after wound scratch. Scale bar is 0.5 mm. (H) Plots show the relative wound confluence of each well up to 44 h after wound scratch. (I) the area under the curve of (H) and (J) final relative wound confluence at 44 h of (H). (I, J) Each data point corresponds to a single well (technical replicate) of *n* = 3. (K, L) Cell viability of MKN‐74 WT and HS1BP3 KO clones (1.1. and 2.4) cells, measured by the MTT metabolic assay (K, *n* = 3) and Trypan blue assay (L, *n* = 2). (B–D, I–L) The data plotted are mean ± standard deviation (B–D) or standard error (I–L) of the mean. (B, C, H) All P values were determined using one‐way ANOVA with Dunnett's multiple comparison test. *P < 0.05; **P < 0.01; ***P < 0.001; ****P < 0.0001; n.s. = not significant.

The protein level of HS1BP3 was significantly highest in MKN‐74 cells (Fig. 2A,B), which also had the highest level of TKS5 (Fig. 2A,C), while cortactin levels did not differ among the three cell lines (Fig. 2A,D). Intriguingly, a correlation between HS1BP3 and TKS5 expression was also found when studying co‐expression among the genes in TCGA and GTEx datasets and in Kaplan–Meier plots. mRNA expression of *HS1BP3* showed a significant and strong positive correlation with the expression of *SH3PXD2A*, the gene encoding TKS5 (Fig. S2A), while HS1BP3 negatively correlated with *CTTN*, the gene encoding cortactin (Fig. S1). In Kaplan–Meier plots, high *SH3PXD2A* levels predicted poorer outcomes for gastric cancer patients but not for TNBC patients (Fig. S2C,D), while *CTTN* expression levels did not predict survival outcomes of neither gastric nor TNBC patients (Fig. S2E,F). Together this indicates a positive correlation between HS1BP3 and TKS5 expression.

To further understand the role of HS1BP3 in tumorigenesis, we generated HS1BP3 knockout (KO) MKN‐74 cells using two different CRISPR/Cas9 guide RNAs targeting either exon 1 or 2 (guide 1 and guide 2, respectively) of the HS1BP3 gene. One cell clone per guide was selected for further experiments, both showing efficient depletion of HS1BP3 (guide 1 clone 1 = 1.1; and guide 2 clone 4 = 2.4) (Fig. 2E). The KO efficiency was further assessed by GST pulldown experiments with the SH3 domain of cortactin, showing strong binding of HS1BP3 using cell lysates from MKN‐74 wild‐type (WT) cells, while almost no HS1BP3 was detected in cell lysates from KO clone 2.4 and only a small amount of HS1BP3 was present in KO clone 1.1 (Fig. 2F). Using a wound‐healing assay to study directed cell migration, we observed a significantly reduced level of wound closure in both HS1BP3 KO clones compared to MKN‐74 WT cells (Fig. 2G–J). Intriguingly, the effect was stronger in clone 2.4 than in clone 1.1, in line with the better HS1BP3 KO efficiency in clone 2.4. It should be noted that the MKN‐74 cells migrate slowly, as the wound did not close completely within 50 h of culturing, and a difference in migration between WT and KO cells was only observed after 20 h. Hence, it is likely that a difference in proliferation, rather than migration, explains the observed difference between MKN‐74 WT and HS1BP3 KO cells in the wound‐healing assay. This was further confirmed by a loss of difference between WT and HS1BP3 KO cells in proliferation and wound closure when treating the cells with the antiproliferative agent Mitomycin C [32] (Fig. S3A–D). To our surprise, both HS1BP3 KO clones were significantly associated with approximately 15% higher metabolic activity and no change in viability compared with WT cells, as analysed in MTT and trypan incorporation assays respectively (Fig. 2K,L), indicating that the reduced proliferation and wound closure of HS1BP3 KO cells were not due to increased cell death.

### 3.3 HS1BP3 directly interacts with cortactin via its third proline‐rich region PRR3.1

**Fig. 3:**
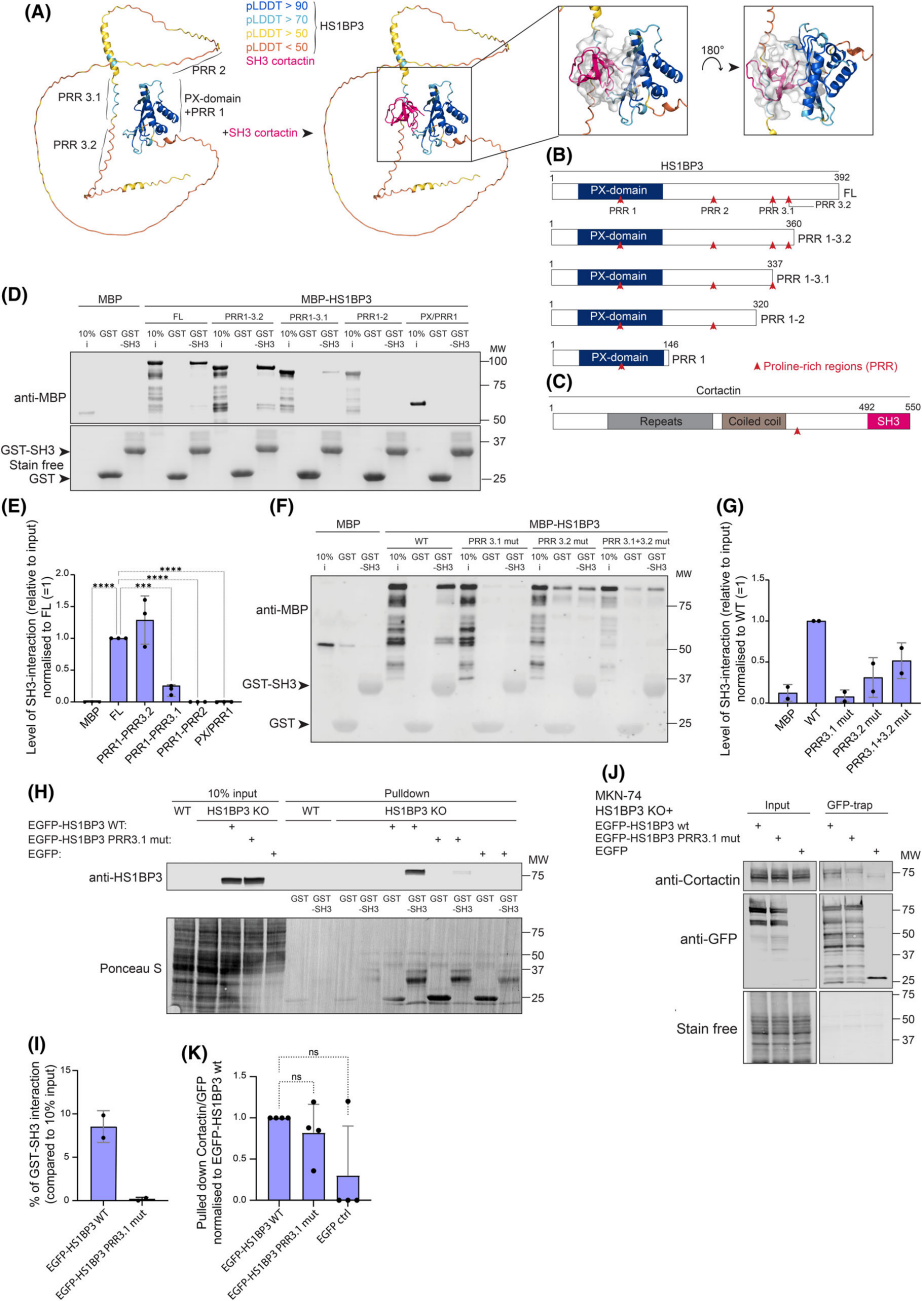
HS1BP3 Interacts directly with the SH3‐domain of cortactin via its third proline‐rich region PRR3.1. (A) An AlphaFold‐generated prediction on the interaction between HS1BP3 and the SH3 domain of cortactin. The per‐residue measures of local confidence (Plddt) values are scaled 0–100 and indicate confidence of the predictions. The cloud indicates interaction surfaces. (B) The cartoon shows the Full length (FL) HS1BP3 and position of the HS1BP3 PX domain and the proline‐rich regions (PRR). The C‐terminal deletion mutants used to map the interaction of HS1BP3 with the SH3 domain of cortactin are shown. (C) The cartoon indicates the F‐Actin binding repeats region, the coiled coil, PRR and SH3 domain of cortactin. (D) GST‐SH3 pulldown assay using recombinant GST, GST‐SH3 from cortactin, and MBP‐HS1BP3 full length and deletion mutants, followed by immunoblot analysis of bound HS1BP3 proteins using an anti‐MBP antibody. GST and GST‐SH3 were visualised using stain‐free gels. 10% of the input (I) MBP protein was loaded. *n* = 3. (E) Quantification of the data in (D), showing the mean level of HS1BP3 interaction to GST‐SH3. (F) GST‐pulldown assay of recombinant MBP HS1BP3 full‐length WT and mutants of PRR3.1 and/or PRR3.2, followed by immunoblot analysis of bound HS1BP3 proteins using an anti‐MBP antibody. The levels of GST and GST‐SH3 used are seen. 10% of the input (i) MBP protein was loaded. *n* = 2. (G) Quantification of the data in (F) showing the mean level of HS1BP3 interaction to GST‐SH3. (H) GST‐pulldown assay of cell lysates from MKN‐74 WT cells or HS1BP3 KO cells rescued or not with EGFP‐HS1BP3 WT, EGFP‐HS1BP3 PRR3.1‐mutant or EGFP‐control, followed by anti‐HS1BP3 immunoblot analysis to determine binding to GST and GST‐SH3 (SH3 domain of cortactin), *n* = 2. (I) Quantification of the data in (H), showing the mean percentage EGFP‐HS1BP3 binding to GST‐SH3, *n* = 2. The levels of GST and GST‐SH3 were detected by Ponceau S staining of the membrane. (J) Cell lysates from MKN‐74 cells expressing EGFP HS1BP3 WT, PRR3.1‐mutant or EGFP‐control were subjected to GFP‐trap pulldown and immunoblot analysis of cortactin, *n* = 4. (K) quantification of (J) showing the mean percentage pulldown of cortactin relative to pulled down GFP. (E, G, I, K) Data are plotted as mean ± standard error of the mean. *P*‐values were determined using one‐way ANOVA with Dunnett's multiple comparison test. *P; **P < 0.01; ***P < 0.001; ****P < 0.0001; n.s. = not significant.

Previous publications report that stable overexpression of cortactin in cancer cell lines is associated with an invasive phenotype [9, 10]. Although *CTTN* expression levels did not predict survival outcomes of gastric cancer patients (Fig. S2E), the interaction of HS1BP3 with cortactin may be important for HS1BP3's role in gastric cancer development. We previously demonstrated that HS1BP3 interacts with the SH3 domain of cortactin [7] but did not determine the HS1BP3 region responsible for its interaction with cortactin. PRRs commonly interact with SH3 domains [11, 33], and we speculated that HS1BP3 binds to cortactin's SH3 domain via one or more of its PRRs. To address this, we first generated HS1BP3‐SH3 docking models using the AlphaFold Server (Fig. 3A). The five resulting models suggested that HS1BP3 interacts with the cortactin SH3 domain via its lipid‐binding PX domain and a region spanning its large C‐terminal PRR3 (Fig. 3A–C). We have previously shown that the PX domain is indispensable for the HS1BP3‐cortactin interaction [7], suggesting limitations of the AlphaFold model. We therefore decided to generate and purify four MBP‐tagged HS1BP3 C‐terminal deletion mutants (Fig. 3B) that were incubated with the SH3 domain of cortactin fused to GST and bound to glutathione beads (Fig. 3D,E). While the full‐length MBP‐HS1BP3 bound strongly to GST‐SH3, the deletion mutants HS1BP3^PRR1^ and HS1BP3^PRR1–2^ containing the PX domain and PRR1 or PRR1‐2, respectively, did not interact with GST‐SH3 (Fig. 3D,E). Since the AlphaFold models indicated that the SH3 domain interaction with the HS1BP3 PRR3 region may vary between two potential PRRs (PRR3.1 and PRR3.2) (Fig. 3A), we decided to make C‐terminal deletion mutants containing either PRR3.1 (HS1BP3^PRR1–3.1^) or PRR3.2 (HS1BP3^PRR1–3.2^) (Fig. 3B). Both these deletion mutants bound to GST‐SH3 but the interaction with HS1BP3^PRR1–3.1^, containing PRR1‐3.1, was strongly reduced (Fig. 3D,E). Overall, our data indicate that the PX domain, PRR1 and PRR2, are dispensable for the direct interaction between HS1BP3 and the SH3 domain of cortactin, while PRR3.1 and PRR3.2 are the likely interaction regions.

To further validate a role for HS1BP3 PRR3 in binding to the SH3 domain of cortactin, we produced full‐length MBP‐HS1BP3 having the prolines of PRR3.1 and PRR3.2 replaced with alanine, either separately or together (Fig. 3F,G). Intriguingly, mutation of PRR3.1 completely prevented the binding between MBP‐HS1BP3 and GST‐SH3. Mutation of PRR3.2 induced nonspecific interactions of both the single and double mutant, indicating it may induce HS1BP3 conformational changes. Since the HS1BP3 PRR3.1 mutation prevented the direct interaction between MBP‐HS1BP3 and GST‐SH3 *in vitro*, we next set out to investigate the role of HS1BP3‐PRR3.1 in cells. The MKN‐74 HS1BP3 KO cells were rescued with EGFP‐tagged WT or PRR3.1‐mutant HS1BP3, followed by incubation of cell lysates with GST or GST‐SH3 (cortactin). Indeed, immunoblot analysis of bound HS1BP3 revealed that binding of the HS1BP3 PRR3.1 mutant to GST‐SH3 was strongly reduced compared to HS1BP3 WT (Fig. 3H,I). Co‐immunoprecipitation of endogenous cortactin with EGFP‐HS1BP3 WT or PRR3.1 mutant demonstrated that the PRR3.1 mutant can still interact with cortactin in cells, but at a reduced level (Fig. 3J,K). We postulate that this may be due to indirect interactions of HS1BP3 with cortactin in a protein complex, since there are at least 221 SH3‐domain containing proteins that can link them together [33].

Taken together, our data indicate that the PRR3 region of HS1BP3 facilitates its interaction with the SH3 domain of cortactin, where mutation of PRR3.1 in full‐length HS1BP3 prevents its direct interaction with cortactin's SH3 domain.

### 3.4 The direct HS1BP3‐cortactin interaction modulates cancer cell proliferation and invasion

**Fig. 4:**
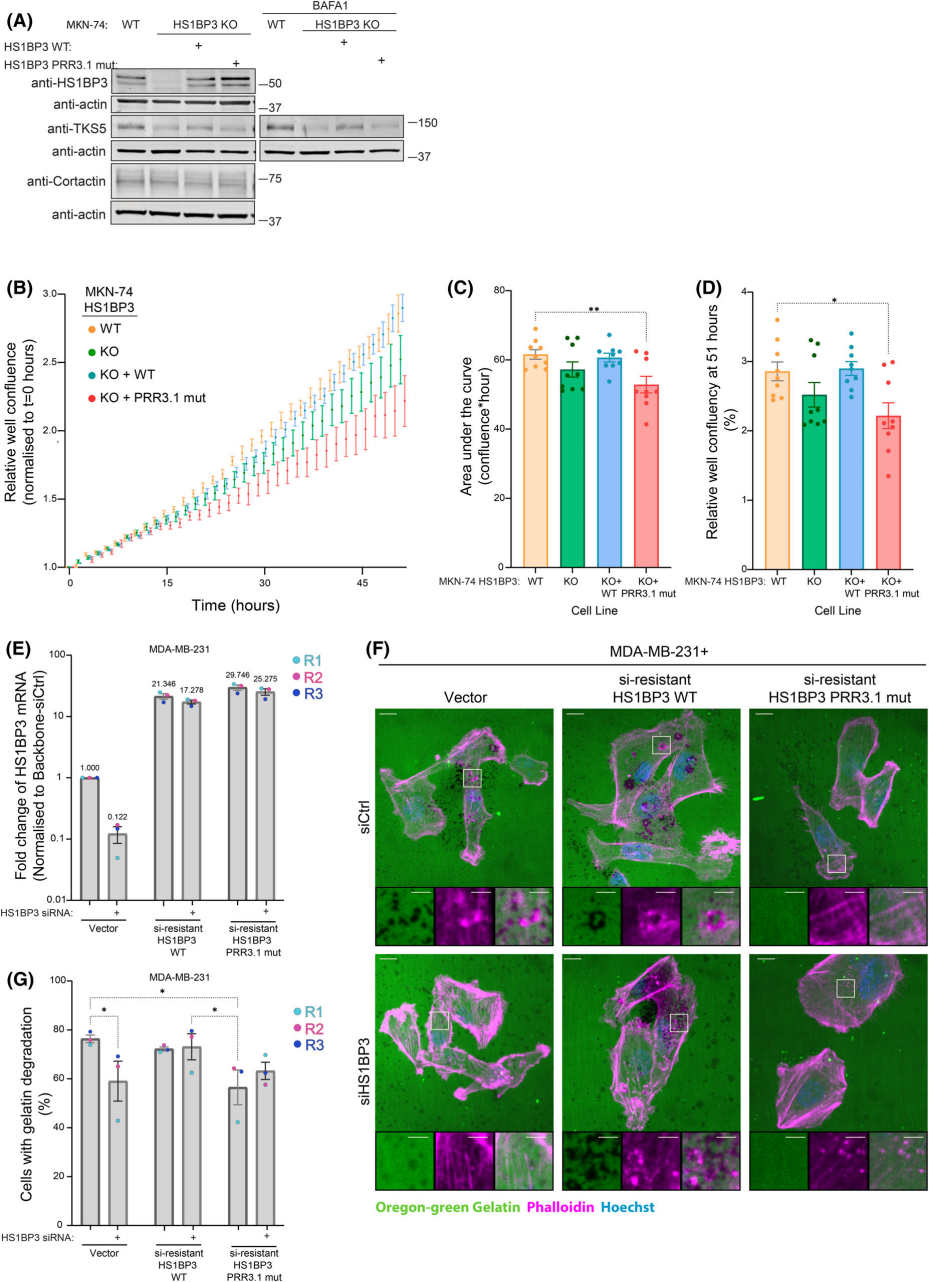
The HS1BP3‐cortactin interaction modulates gastric cell proliferation and invasion in TNBC. (A) Cell lysates from MKN‐74 WT, HS1BP3 KO or HS1BP3 KO with low‐expressing HS1BP3 WT or PRR3.1 mutant were treated or not for 2 h with 100 nm Bafilomycin A1 (BafA1) followed by immunoblot analysis for cortactin and TKS5 levels, *n* = 3. (B) MKN‐74 WT, HS1BP3 KO 2.4 and KO 2.4 with rescue of HS1BP3 WT or PRR.1 mutant were seeded sparsely in 96‐well plates for live cell microscopy to observe their proliferation rate as mean relative confluence every hour over 51 h, *n* = 3. (C, D) Quantifications of (B) showing the area under the curve (C) and relative well confluency after 51 h (D). Each data point corresponds to a single well (technical replicate). (E–G) MDA‐MB‐231 stably expressing negative‐control vector, siRNA‐resistant HS1BP3 WT or PRR3.1 mutant were transfected with negative‐control siRNA or siRNA targeting endogenous HS1BP3. 72 h after transfection the cells were harvested and checked for HS1BP3 expression using quantitative PCR (E) or seeded sparsely on coverslips coated with Oregon‐Green labelled gelatin for 4 h before PFA‐fixation and staining with Hoechst and phalloidin before visualising the cells by confocal imaging (F). Images are taken with a Nikon Ti2‐E microscope with a Yokogawa CSU‐W1 SoRa spinning disc 60 × WI water immersion objective. Scale bar is 9 μm, inset scale bars are 3 μm and inset rectangles are 9 × 9 μm^2^. (E) data are plotted on a Log_10_ scale for easier visualisation and comparison of values. The mean expression value normalised to vector siCtrl are written on top of the bar. Each data point corresponds to one replicate (G) quantification of (F) showing the percentage of quantified cells with observed gelatin degradation spots within cell boundary compared to total number of quantified cells. Each data point corresponds to one replicate quantifying 61–165 cells. (B–E, G) Data are plotted as mean ± standard error of the mean. *P* values were determined using one‐way ANOVA with Dunnett's multiple comparison test (C, D) or two‐way ANOVA with Holm‐Šídák (E) or Fisher's LSD (G) test. *P; **P < 0.01.

As the EGFP‐tagged HS1BP3 WT and PRR3.1‐mutant proteins were expressed at a much higher level than the endogenous HS1BP3 protein (Fig. 3H), we decided to generate new MKN‐74 HS1BP3 KO cells stably expressing untagged HS1BP3 WT and PRR3.1‐mutant at a level close to the endogenous protein (Fig. 4A). These cells, together with MKN‐74 WT and HS1BP3 KO cells, were then used to determine whether the HS1BP3‐cortactin interaction was important for cell proliferation. Intriguingly, HS1BP3 WT but not the PRR3.1 mutant was able to rescue the reduced proliferation of HS1BP3 KO cells (Fig. 4B–D), indicating that the interaction of HS1BP3 with cortactin promotes cell proliferation.

Since HS1BP3 has been found to act as a negative regulator of autophagy and autophagy levels can affect cancer development [3, 7], we aimed to determine whether HS1BP3 affects proliferation of MKN‐74 cells via regulation of autophagy. By quantifying the level of autophagosomes (LC3B puncta) and autolysosomes (LC3B‐LAMP1 overlap) in immunofluorescence images (Fig. S4A–D) and measuring LC3B flux by western blot (Fig. S4E–H) in control and starved conditions, we did, however, not detect any significant differences in autophagy levels in HS1BP3 KO cells versus HS1BP3 WT or PRR3.1 rescue cells. Thus, the interaction of HS1BP3 with cortactin does not seem to regulate autophagy in gastric adenocarcinoma cells.

To further assess whether the HS1BP3‐cortactin interaction may play a role in cancer cell invasion, MKN‐74 cells were seeded on Oregon‐Green labelled gelatin for up to 17 h, followed by imaging analysis. Unfortunately, we did not detect any convincing matrix degradation of the MKN‐74 WT cells (Fig. S5A), suggesting that these cells are not suited for invasion assays.

As high *HS1BP3* expression also was found to correlate with poor survival of patients with TNBC (Fig. 1A, Fig. S1B; HR = 1.92), we decided to use the TNBC cell line MDA‐MB‐231 to assess the role of HS1BP3 in cancer cell invasion. MDA‐MB‐231 is a cell line with adenocarcinomal histology that robustly degrades gelatin [34]. Intriguingly, siRNA‐mediated depletion of HS1BP3 in MDA‐MB‐231 cells (Fig. 4E) caused a significant reduction in the number of cells with areas of gelatin degradation after 4 h of incubation on Oregon‐Green Gelatin compared to cells transfected with control siRNA (Fig. 4F,G). The gelatin degradation was rescued in cells with stable expression of siRNA‐resistant HS1BP3 WT but not in cells expressing siRNA‐resistant HS1BP3 PRR3.1‐mutant upon knockdown of HS1BP3 (Fig. 4F,G). Taken together, our data demonstrate that the interaction of HS1BP3 with cortactin is important for cancer cell proliferation and invasion.

### 3.5 The HS1BP3‐cortactin interaction regulates levels of TKS5‐associated multivesicular endosome positioning and secretion

To further understand how the HS1BP3‐cortactin interaction modulates cancer development, we decided to follow up on our initial finding of a correlation between HS1BP3 and TKS5 expression levels in gastric cancer cell lines (Fig. 2A). Intriguingly, we observed a reduced level of TKS5 in the MKN‐74 HS1BP3 KO cells, which was rescued by HS1BP3 WT but not the PRR3.1‐mutant (Fig. 4A). TKS5 did not accumulate upon treatment with the lysosomal V‐ATPase inhibitor Bafilomycin A1 (Fig. 4A), indicating that cells lacking HS1BP3 or being unable to interact with cortactin may have lower levels of TKS5 expression. Indeed, the mRNA level of *SH3PXD2A* (TKS5) was reduced in MKN‐74 HS1BP3 KO cells compared with WT and HS1BP3 WT rescue cells (Fig. S5B). In contrast, HS1BP3 KO and WT rescue did not affect cortactin mRNA levels (Fig. S5C), in line with the expression‐correlation patterns described above (Fig. 2A; Fig. S2A,B). Overall, our data suggest that the direct interaction of HS1BP3 with cortactin may affect TKS5 protein levels.

**Fig. 5:**
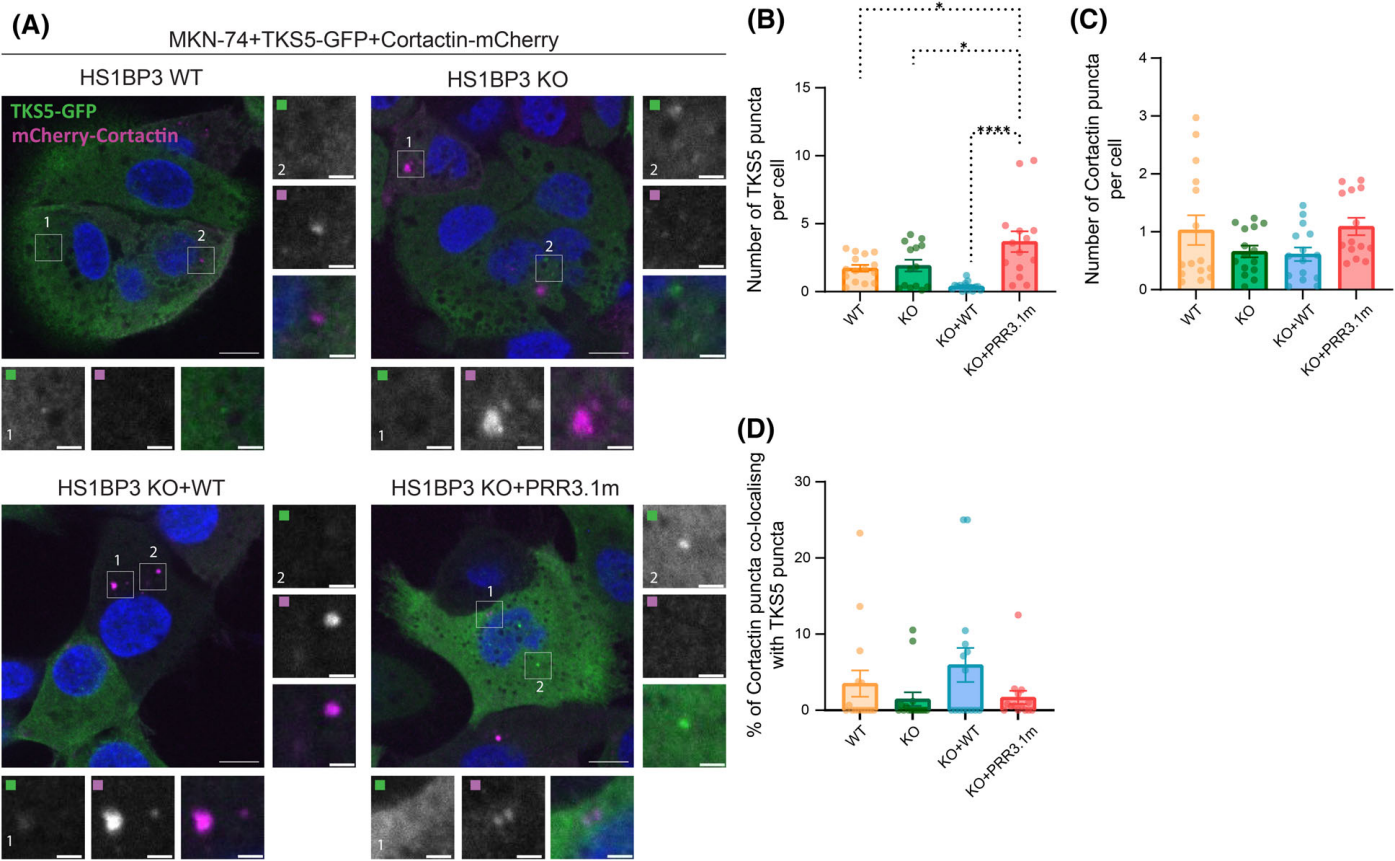
Ablation of the HS1BP3‐cortactin interaction is associated with increased levels of TKS5 puncta. (A) representative fluorescence imaging of overexpressed TKS5‐GFP and mCherry‐Cortactin in the middle of MKN‐74 HS1BP3 WT, KO with or without rescue of HS1BP3 WT or PRR3.1. Images were acquired with a Nikon CREST X‐Light V3 spinning disc microscope using a 60× oil objective (NA 1.42). Scale bars: 10 μm (main figure) or 2 μm (inset). (B–D) quantification of (A) representing the number of TKS5 puncta per cell (B), the number of cortactin puncta per cell (C) and the percentage cortactin overlapping with TKS5 (D). Each data point corresponds to a single field of view. Data are mean ± Standard error of the mean, the statistical significance was calculated with ordinary one‐way ANOVA, followed by Tukey's multiple comparison test (*n* = 4, 103–238 cells were quantified in each condition). *P; ****P < 0.0001.

To determine whether HS1BP3 may regulate the cortactin‐TKS5 interaction, MKN‐74 WT and HS1BP3 KO cells were transfected with TKS5‐EGFP, followed by GFP pulldown and immunoblot analysis for cortactin and HS1BP3, showing that HS1BP3 neither affected the TKS5‐cortactin interaction nor interacted with TKS5 (Fig. S5D,E). We then postulated that HS1BP3 may modulate the subcellular localisation of cortactin and/or TKS5. Hence, MKN‐74 WT and HS1BP3 KO cells, rescued or not with HS1BP3 WT or the PRR3.1‐mutant, were stably transduced with TKS5‐GFP and mCherry‐cortactin. The cells were analysed by fluorescence microscopy for the colocalisation and/or changes in TKS5 and cortactin puncta (Fig. 5). It is well‐established that invasive cell lines that degrade extracellular matrix form ventral invadopodia when grown on glass coverslips due to cell matrix secretion [17, 34]. In line with MKN‐74 cells not being invasive when grown on gelatin (Fig. S4A), we were unable to detect any TKS5‐GFP puncta ventrally. However, we observed a significant increase in the number of TKS5‐GFP puncta in the middle of the cell in the HS1BP3 KO MKN‐74 cells rescued with the HS1BP3 PRR3.1‐mutant compared with WT HS1BP3 (Fig. 5A,B). While we did not observe any significant difference in the number of mCherry‐cortactin puncta per cell between the MKN‐74 genotypes (Fig. 5C), there was a trend of decreased TKS5‐GFP colocalisation with mCherry‐cortactin in MKN‐74 HS1BP3 KO and PRR3.1 rescue cells compared to WT cells and HS1BP3 KO cells rescued with HS1BP3 WT (Fig. 5D).

**Fig. 6:**
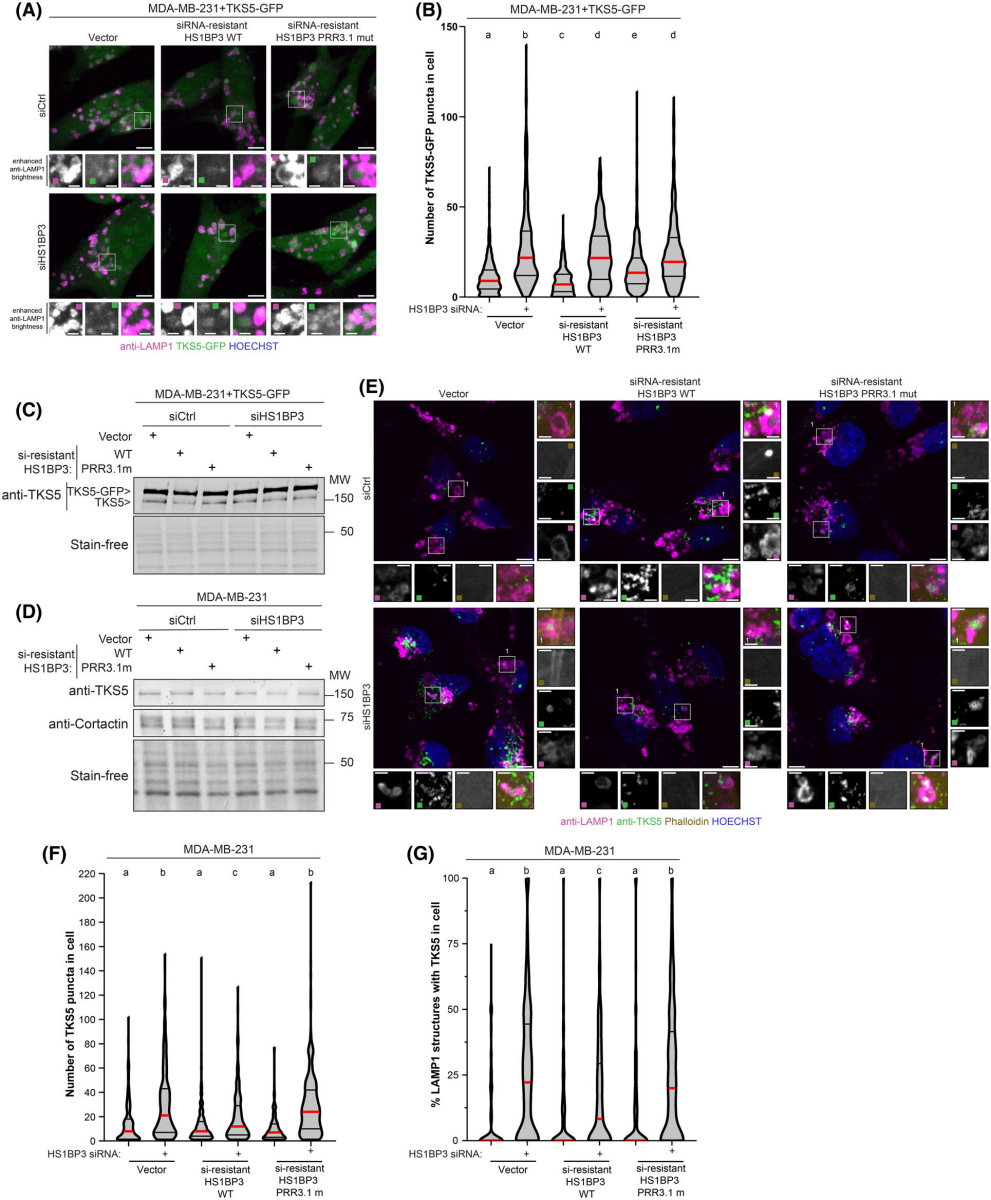
The HS1BP3‐cortactin interaction regulates levels of TKS5‐associated multivesicular endosome (A) Representative maximum intensity projection (immunofluorescence images of MDA‐MB‐231 with stable expression of TKS5‐GFP with a negative control vector, siRNA‐resistant HS1BP3 WT or PRR3.1‐mutant. The cells were subjected to transient transfection with a negative siRNA‐control or siRNA targeting HS1BP3 and were PFA‐fixed after 72 h of knockdown. This was followed by immunofluorescence staining targeting anti‐LAMP1. Images are taken with a Nikon Ti2‐E microscope with a Yokogawa CSU‐W1 SoRa spinning disc 60xWI water immersion objective. Scale bar and insets are 5 μm, scale bar in inset is 1.7 μm. The insets are shown with brighter anti‐LAMP1 channel to highlight the localisation of TKS5‐GFP in LAMP1‐structures out of focus. *n* = 3. (B) violin plot with quantification of (A) number of segmented TKS5‐GFP puncta in all segmented cells. 44–660 cells were quantified in each condition. (C) Cells treated as in (A) were lysed instead of PFA‐fixed and total TKS5 (TKS5‐GFP and endogenous) were analysed by western blot with total protein level (stain‐free) as a loading control. Representative of three experiments. (D) MDA‐MB‐231 cells with negative control vector or siRNA resistant HS1BP3 WT or PRR3.1‐mutant were subjected to transient transfection with a negative siRNA‐control or siRNA targeting HS1BP3 and were lysed after 72 h of knockdown. Total TKS5, cortactin and total protein (stain‐free) were analysed by western blot with total protein level (stain‐free) as a loading control. (E) Representative immunofluorescence images of cells treated as in (D) but PFA‐fixed after 72 h of knockdown followed by antibody staining with anti‐TKS5 and anti‐LAMP1, and imaging. Images are taken with a Nikon Ti2‐E microscope with a Yokogawa CSU‐W1 SoRa spinning disc 60 × WI water immersion objective. Inset is 5.5 × 5.5 μm, and scale bar 5.5. Inset scale bars are 1.8 μm. *n* = 3 (F, G) violin plots with quantifications of (E) showing the (F) quantified number of TKS5 in cells and (G) the % of LAMP1 structures in cells that contain TKS5. In (B, F, G) the thick red line is the median and the black horizontal lines are the quartiles. 41–191 cells were quantified in each condition. (B, F, G) Significant difference (*P* < 0.05) is indicated on basis of different letter (a–e) between the condition (in conditions with the same letter, the difference between the means is not statistically significant). The statistical significance was calculated with ordinary one‐way ANOVA followed by Holm‐Šídák multiple comparison test.

Since we observed an increase in the number of TKS5‐GFP puncta in the MKN‐74 PRR3.1 rescue cells (Fig. 5B) with few colocalising with cortactin, we postulated that these puncta may represent TKS5 in multivesicular endosomes, from where TKS5 is secreted with metalloproteases important for matrix degradation [25, 35]. To investigate this, invasive MDA‐MB‐231 cells stably overexpressing TKS5‐GFP were immunolabelled for LAMP1 and subjected to immunofluorescence microscopy analysis, demonstrating a consistent localisation of TKS5‐GFP in LAMP1‐positive structures (Fig. 6A). We did not observe any TKS5‐GFP puncta outside LAMP1‐positive structures. The fact that the TKS5‐GFP signal seems not to be quenched by the low pH of lysosomes indicates that TKS5‐GFP is found within the intraluminal vesicles of LAMP1‐positive multivesicular endosomes. Indeed, staining for the multivesicular body (MVB) marker CD63 confirmed the localisation of TKS5‐GFP and endogenous TKS5 in MVBs in MDA‐MB‐231 cells (Fig. S6A,B) and to a lesser degree TKS5‐GFP in MKN‐74 cells (Fig. S6C). Intriguingly, while overexpression of HS1BP3 WT significantly decreased the occurrence of TKS5‐GFP puncta, overexpression of the PRR3.1 mutant or siRNA‐mediated knockdown of HS1BP3 significantly increased the number of TKS5‐puncta (Fig. 6A,B), in line with our findings in MKN‐74 cells (Fig. 5A,B). The increased number of TKS5‐puncta observed in HS1BP3‐depleted cells was significantly reduced by overexpression of siRNA‐resistant HS1BP3 WT or the PRR3.1 mutant (Fig. 6A,B). It is interesting to note that the changes in TKS5‐GFP puncta levels occurred without affecting the total TKS5‐GFP or endogenous TKS5 levels, as observed by western blot (Fig. 6C).

To test whether HS1BP3 expression levels also have similar effects on endogenous TKS5, MDA‐MB‐231 cells with or without expression of siRNA‐resistant HS1BP3 WT or PRR3.1 mutant were depleted or not of HS1BP3, followed by immunoblotting analysis. Similar to overexpressed TKS5‐GFP (Fig. 6C), the total levels of endogenous TKS5 did not change between the conditions (Fig. 6D). The cells were then fixed and stained for endogenous TKS5 with LAMP1 to further assess the recruitment of TKS5 to MVBs (Fig. 6E). Although endosomal localisation of endogenous TKS5 is a rare event compared to overexpressed TKS5‐GFP [25, 36], we observed a significant increase of TKS5 puncta per cell in HS1BP3‐depleted cells compared with WT MDA‐MB‐231 cells, which was reduced by rescue with WT HS1BP3 but not by the PRR3.1‐mutant (Fig. 6E,F). The endogenous TKS5 puncta were cytoplasmic and did not appear in F‐actin‐rich puncta as observed by phalloidin stain (Fig. 6E), suggesting they were different from invadopodia. We also observed the presence of TKS5 signal in 10% of LAMP1‐labelled late endosomes/lysosomes (Fig. 6G). Importantly, HS1BP3 knockdown significantly increased the percentage of LAMP1‐positive structures containing TKS5 in WT cells and in cells expressing the HS1BP3 PRR3.1‐mutant (Fig. 6G).

**Fig. 7:**
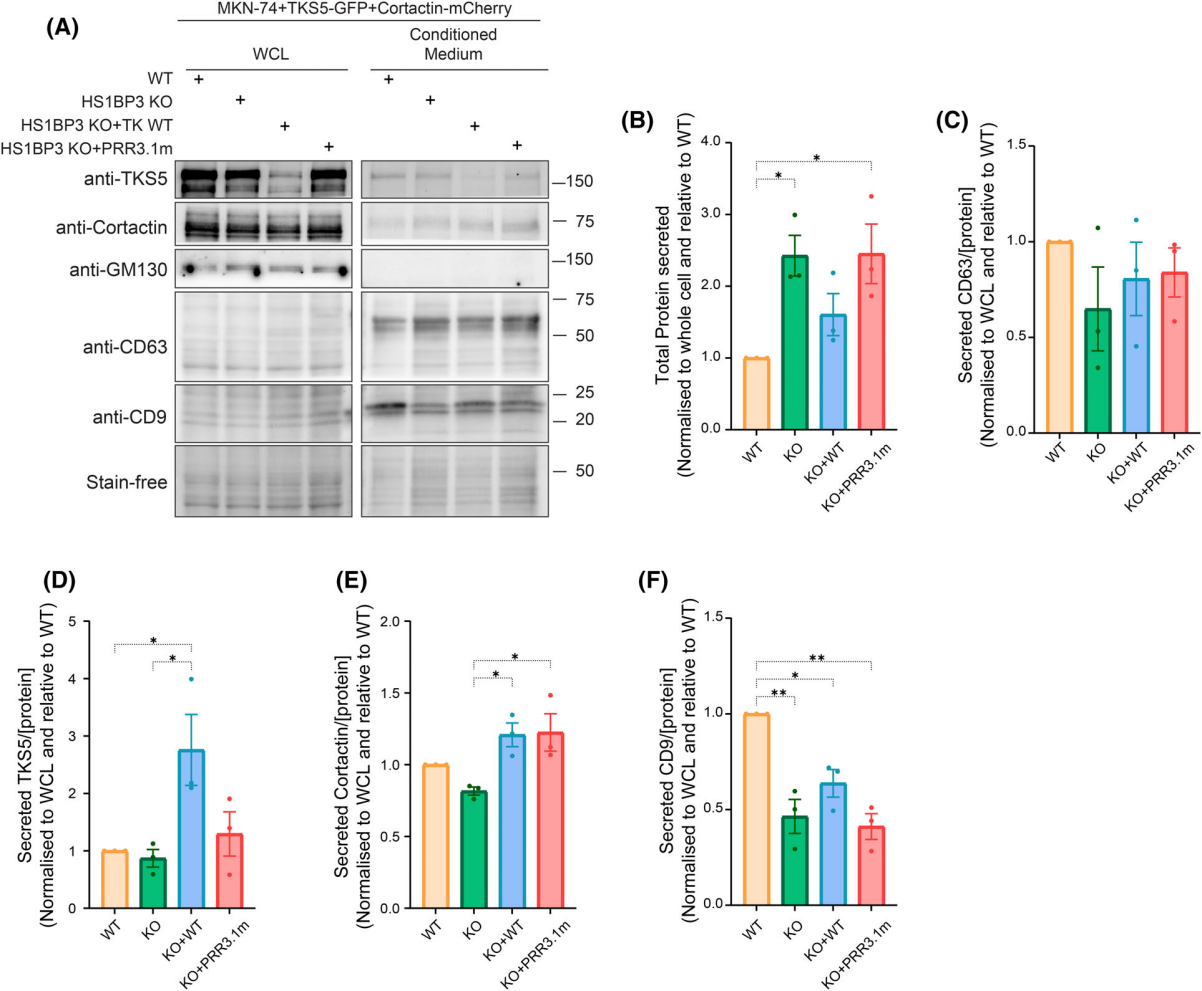
The HS1BP3‐cortactin interaction regulates secretion levels and type of extracellular vesicles secreted (A) MKN‐74 cells with HS1BP3 WT and KO 2.4 with HS1BP3 WT and PRR3.1 mutant were seeded in 150 mm dishes and serum‐starved for 44 h before the conditioned media was collected and extracellular vesicles concentrated. 10% of the whole cell lysate (WCL) and 50% of the concentrated media were analysed by western blot with indicated antibodies and total protein level (stain‐free) as loading control. *N* = 3 (B) quantitation of the total protein (stain‐free) secreted compared to the total protein in the whole cell lysate (WCL). (C–F) quantitation of the secretion of the indicated proteins normalised to the amount of total protein secreted. The values were further normalised to the WCL levels before plotting relative values according to the WT condition. (B–F) Data are mean ± Standard error of the mean and the statistical significance was calculated with ordinary one‐way ANOVA followed by Tukey's multiple comparison test. *P < 0.05; **P < 0.01.

Since the number of TKS5‐GFP puncta was strongly increased in MKN‐74 and MDA‐MB‐231 cells expressing the HS1BP3 PRR3.1 mutant (Fig. S5A,B; Fig. 6B,F), we speculated that this may be associated with changes in the secretion of TKS5 in MVBs, as cortactin is known to promote secretion of CD63‐positive MVBs [16, 17, 18, 37, 38]. To test this, we collected conditioned media from MKN‐74 WT cells and from *HS1BP3* KO cells with or without HS1BP3 WT or PRR3.1 overexpression and used 10 kDa cut‐off spin‐columns to enrich the media of extracellular vesicles (Fig. 7). Strikingly, we observed that loss of HS1BP3 significantly increased general protein secretion compared to WT cells and that this was rescued with the expression of HS1BP3 WT but not with the PRR3.1 mutant (Fig. 7A,B). Intriguingly, the secreted level of CD63 corresponded to the overall amount of protein secreted in the different cell lines (Fig. 7A–C), suggesting that HS1BP3 may function as a negative regulator of the secretion of CD63‐positive exosomes, and that this requires its interaction with cortactin. From these data, we did not observe a clear relationship between the level of TKS5 or cortactin secretion upon HS1BP3 KO or rescue with the WT or PRR3.1 mutant (Fig. 7D,E). Although our assay does not distinguish between different types of extracellular vesicles, it is interesting to note an opposite effect of HS1BP3 levels on CD9 secretion, a protein that can be found in a different extracellular vesicle subpopulation than CD63 [39]. We observed less CD9 being secreted in HS1BP3 KO cells than in WT cells, which was partly rescued with HS1BP3 WT but not the PRR3.1 mutant overexpression (Fig. 7F). Taken together, our data suggest that HS1BP3 and its interaction with cortactin modulate the intracellular sorting and secretion of extracellular vesicles and, by extension, cell invasiveness.

## 4 Discussion

Here, we demonstrate that an interaction between HS1BP3 and cortactin modulates cell proliferation and invasion, providing an explanation to the observed correlation between high HS1BP3 levels and poor survival of gastric adenocarcinoma and TNBC patients. Gastric cancer is currently the 5th most common cancer type and is attributable to 8.3% of cancer deaths worldwide [40, 41, 42]. Most gastric cancer patients are only diagnosed when the cancer has reached an advanced stage, and with few effective treatments other than surgery, the prognosis remains poor [43]. Similarly, breast cancer is the topmost cause of cancer death in women [44] with the TNBC subtype being the most lethal, with no viable treatments [31]. Hence, it is important to investigate the molecular mechanisms underlying these diseases for earlier detection and development of new responsive therapies.

We have previously shown that HS1BP3 functions as a negative regulator of autophagy and that it interacts with cortactin [6, 7, 8]. To elucidate the molecular mechanisms underlying the negative correlation between HS1BP3 mRNA levels and survival time in gastric adenocarcinoma and in TNBC patients, we first pinpointed the interaction site between HS1BP3 and cortactin and generated an HS1BP3 mutant (PRR3.1 mutant) that abolished the interaction. Intriguingly, we demonstrated that the HS1BP3‐cortactin interaction is important for efficient extracellular matrix degradation in the invasive TNBC cell line MDA‐MB‐231 and for cell proliferation in the noninvasive gastric adenocarcinoma cell line MKN‐74.

High cortactin levels have previously been associated with worsened cancer phenotypes [16, 17, 18, 37, 45], but we detected no significant differences in the survival of high and low‐expressing *CTTN* gastric adenocarcinoma and TNBC patients. Thus, it is possible that cortactin may not be transcriptionally regulated but has a function in cancer development through its interaction with HS1BP3 [7]. Comparing pairs of control and tumour tissue from gastric adenocarcinoma patients, we found that *HS1BP3* is specifically upregulated in the tumour tissue, suggesting that *HS1BP3* is transcriptionally upregulated upon tumorigenesis. To our surprise, we did not detect any significant differences in basal or starvation‐induced autophagy in HS1BP3 KO cells compared to WT MKN‐74 cells, suggesting that the effect of HS1BP3 on proliferation and invasion is independent of autophagy. Since we have previously identified HS1BP3 as a negative autophagy regulator in U2OS, HeLa and HEK293 cells [6, 7, 8] and *in vivo* in Zebrafish [7], autophagy flux in metastatic cancer sites from where MKN‐74 cells are isolated may be regulated differently.

To further understand how the HS1BP3‐cortactin interaction modulates matrix degradation and proliferation, we investigated whether the expression level and/or cellular localisation of the cortactin‐binding protein TKS5 was affected in cells expressing the HS1BP3 PRR3.1 mutant. This was based on the observed co‐expression between *HS1BP3* and *SH3PXD2A*, the gene encoding TKS5 in gastric tissues. TKS5 is a known interactor of cortactin in the initiation of invadopodia and podosome formation for invasion of the extracellular matrix [14, 46]. Loss of HS1BP3 had no effect on the interaction between TKS5 and cortactin, suggesting that HS1BP3 KO cells still can initiate invadopodia formation. However, we observed bright TKS5‐GFP puncta significantly accumulating in cells expressing the HS1BP3 PRR3.1 mutant in both MKN‐74 and MDA‐MB‐231 cells. We further demonstrated that these puncta, as well as endogenous TKS5 puncta, colocalised with LAMP1‐ and CD63‐positive late endosomes/MVBs. It was recently suggested that TKS5 is degraded by autophagy due to the observation of TKS5‐GFP in LAMP1‐positive structures [14]. However, as the low pH of the endolysosomes would have quenched the GFP signal, it is more likely that TKS5 is found in vesicles inside of MVBs. In line with this, we did not observe an accumulation of TKS5 when treating MKN‐74 cells with the lysosomal inhibitor Bafilomycin A1, suggesting that TKS5 is not a cargo for autophagy. Furthermore, there was no significant effect on autophagy levels when knocking down TKS5 [6, 8]. Intriguingly, we found that secretion of CD63 was increased in *HS1BP3* KO cells and in KO cells rescued with the PRR3.1 mutant compared to WT cells. The effect of HS1BP3 on TKS5 secretion was less clear. Thus, the buildup of TKS5 in multivesicular bodies may relate to a prevention of invadopodia formation or other TKS5 interactions required for cell proliferation and invasion.

In line with a build‐up of TKS5 in MVBs in HS1BP3 PRR3.1 mutant cells, regulation of cortactin levels has been associated with changed secretion and matrix degradation [16, 17, 18, 37]. Hence, inhibition of the HS1BP3‐cortactin interaction may reduce matrix degradation by (1) a failure to initiate invadopodia due to increased MVB localisation of TKS5 and/or (2) reduced secretion of autocrine factors important for degradation. The effect of the HS1BP3‐cortactin interaction on cell proliferation may also be explained by a shift in the cells' focus from proliferation to increased metabolic rate, as observed by the MTT assay, and secretion in a more differentiated state. Additionally, or alternatively, a change in the type of vesicles secreted may have an effect on proliferation in the HS1BP3 PRR3.1 cells since we observed that the HS1BP3‐cortactin interaction inversely regulated the secretion of CD9 and CD63. Weaver et al [45] demonstrated that secreted factors from cortactin‐overexpressed cells can rescue reduced proliferation in cortactin‐depleted cells, suggesting that cortactin facilitates secretion of autocrine factors important for proliferation. It is not clear what type of secretion is responsible for the proliferation and invasion effects observed in HS1BP3 PRR3.1 cells. Integrating HS1BP3 and cortactin, we have previously shown that HS1BP3 localises on recycling endosomes [7], which is also the case for cortactin [15].

## 5 Conclusions

With this study, we established the importance of HS1BP3 and its interaction with cortactin in cancer cell malignancy. Abolishing the interaction lowers the proliferation and matrix degradation of gastric adenocarcinoma and TNBC cancer cells and may be explained by the interaction regulating extracellular vesicle secretion in the cells.

## Conflict of interest

The authors declare no conflict of interest.

## Author contributions

AAL was involved in conceptualization, formal analysis, investigation, methodology, project administration, validation, visualisation, and writing – original draft, review, and editing. KS was involved in Conceptualization, formal analysis, investigation, methodology, project administration, writing – review and editing. CV was involved in investigation, formal analysis, writing – review and editing. LTM was involved in investigation, writing – review and editing. NA was involved in investigation, writing – review and editing. RG was involved in resources, writing – review and editing. LGL was involved in validation, writing – review and editing. HK was involved in investigation, writing – review and editing. LE was involved in resources, writing – review and editing. AS was involved in supervision, conceptualization, methodology, project administration, funding acquisition, writing – review and editing.

## Supporting information


**Table S1.** Reagents and tools.
**Fig. S1.** HS1BP3 levels is predictive for patient outcomes in breast and ovarian cancer.
**Fig. S2.** HS1BP3 and SH3PXD2A (TKS5) gene expressions correlate in gastric tissues.
**Fig. S3.** HS1BP3 regulates the proliferation of MKN‐74 cells.
**Fig. S4.** HS1BP3 KO or expression of the PRR3.1‐mutant has no effect on autophagy in gastric adenocarcinoma.
**Fig. S5.** HS1BP3 levels do not affect cortactin‐TKS5 protein interaction.
**Fig. S6.** TKS5 localise to CD63‐positive structures.

## Data Availability

This study includes no data deposited in external repositories.
