# Supplementary material for: Interaction of HS1BP3 with cortactin modulates TKS5 localisation, cell secretion and cancer malignancy
Source: Mol Oncol. 2026 Apr 10;20(8):1986–2007. doi: 10.1002/1878-0261.70248 (PMC13399123; doi:10.1002/1878-0261.70248)
Supplement: Supplementary file 1 [file MOL2-20-1986-s001.pdf]

Supplementary table 1 – Reagents and tools

Reagent/Resource	Reference or Source	Identifier or Catalog Number
**Experimental Models**		
AGS	ATCC	CRL-1739
NCI-N87	ATCC	CRL-5822
MKN-74	JC3B	JCRB0255
MKN-74 HS1BP3 KO	This Study	
MKN-74 HS1BP3 KO + TK HS1BP3 WT	This Study	
MKN-74 HS1BP3 KO + TK HS1BP3 PRR3.1mut	This Study	
MKN-74 HS1BP3 KO + CMV EGFP-HS1BP3 WT	This Study	
MKN-74 HS1BP3 KO + CMV EGFP HS1BP3 PRR3.1mut	This Study	
MKN-74 HS1BP3 KO + EGFP	This Study	
MKN-74 + TKS5-GFP + mCherry-Cortactin	This Study	
MKN-74 HS1BP3 KO+ TKS5-GFP + mCherry-Cortactin	This Study	
MKN-74 HS1BP3 KO+ TKS5-GFP + mCherry-Cortactin + TK HS1BP3 WT	This Study	
MKN-74 HS1BP3 KO+ TKS5-GFP + mCherry-Cortactin + TK HS1BP3 PRR3.1mut	This Study	
MDA-MB-231	ATCC	HTB-26
MDA-MB-231 + TK vector	This Study	
MDA-MB-231 + TK HS1BP3 WT (siRNA-resistant)	This Study	
MDA-MB-231 + TK HS1BP3 PRR3.1mut (siRNA-resistant)	This Study	
MDA-MB-231 + TKS5-GFP	Kindly provided by Camilla Raiborg [25]	
MDA-MB-231 + TKS5-GFP + TK vector	This Study	
MDA-MB-231 + TKS5-GFP + TK HS1BP3 WT (siRNA-resistant)	This Study	
MDA-MB-231 + TKS5-GFP + TK HS1BP3 PRR3.1mut (siRNA-resistant)	This Study	
Pairwise gastric tissue control and gastric adenocarcinoma from 28 patients	This Study	
HEK293FT	Clontech (A.H.-diagnostics AS)	cat# 632180
**Recombinant DNA**		
pGEX kG h-cortactin SH3 (GST-SH3)	kindly provided by J.K. Burkhardt	
pGex 5x-3 (GST)	GE Healthcare	
pEntr1A HS1BP3 WT	[7]	
pEntr1A HS1BP3 (1-320)	This study	
pEntr1A HS1BP3 (1-337)	This study	
pEntr1A HS1BP3 (1-360)	This study	
pTH1 (MBP)	Invitrogen	
pTH1 MBP-HS1BP3	[7]	
pTH1 MBP-HS1BP3 (1-146)	[7]	
pTH1 MBP-HS1BP3 (1-320)	This study	
pTH1 MBP-HS1BP3 (1-337)	This study	
pTH1 MBP-HS1BP3 (1-360)	This study	

pEntr1A HS1BP3 PRR3.1mut (P322A, P324A, P326A, P330A, P332A, P333A)	This study	
pEntr1A HS1BP3 PRR3.2 mut (P338A, P341A, p344A, P347A, P348A)	This study	
pEntr1A HS1BP3 PRR3.1 mut + PRR3.2 mut	This study	
pTH1-HS1BP3 PRR3.1mut (P322A, P324A, P326A, P330A, P332A, P333A)	This study	
pTH1-HS1BP3 PRR3.2mut (P338A, P341A, p344A, P347A, P348A)	This study	
pTH1-HS1BP3 PRR3.1mut (P322A, P324A, P326A, P330A, P332A, P333A) + PRR3.2 mut ((P338A, P341A, p344A, P347A, P348A)	This study	
pLVX (CMV) Zeocin-resistant	Kindly provided by Alf Lystad	
pDEST-EGFP-SNX18	[21]	
pLVX (CMV) EGFP-HS1BP3 WT	This study	
pLVX (CMV) EGFP-HS1BP3 PRR3.1mut	This study	
pLENTI-III-PGK (puro)-EGFP	Kindly provided by Alf Lystad	
pLVX (TK) Zeocin-resistant	Kindly provided by Alf Lystad	
pLVX (TK) HS1BP3 WT	This study	
pLVX (TK) HS1BP3 PRR3.1mut	This study	
pLVX (TK) HS1BP3 WT siHS1BP3-resistant	This study	
pLVX (TK) HS1BP3 PRR3.1mut siHS1BP3 resistant	This study	
pEGFP-N1	Clontech	
pEGFP-TKS5-N1	Kindly provided by Camilla Raiborg	
pCDH-PGK-TKS5-GFP	Kindly provided by Camilla Raiborg	
pLenti-III-PGK-mCherry-Cortactin	Kindly provided by Camilla Raiborg	
psPAX2	Kindly provided by Didier Trono - Addgene	cat# 12260
pCMV-VSVG	Kindly provided by Bob Weinberg – Addgene [27]	cat# 8454
pSpCas9(BB)-2A-Puro (PX459)	Kindly provided by Feng Zhang – Addgene [26]	cat# 48139
PX459 HS1BP3-CRISPRS/Cas9-guide 1	This Study	
PX459 HS1BP3-CRISPRS/Cas9-guide 2	This Study	
**Antibodies**		
SH3PXD2A (TKS5). WB: 1:1,000, IF: 1:400	Sigma Aldrich	cat# HPA037923
Cortactin ((p80/85) Antibody, clone 4F11). WB: 1:1,000	Millipore (Upstate)	cat# 05-180
Cortactin [EP1922Y]. WB: 1:1,000	Abcam	cat# ab81208
HS1BP3 (- C-terminal). WB: 1:1,000	Abcam	cat# ab200592
B-Actin ((8H10D10) Mouse Ma). WB: 1:1,000	Cell Signaling	cat#3700
B-Actin ((13E5) Rabbit mAb). WB: 1:1,000	Cell Signaling	cat#4970
LC3 ((D11) XP® Rabbit mAb). WB: 1:1,000	Cell Signaling	cat# 3868S
GST (HRP Anti-GST antibody). WB: 1:10,000	Abcam	cat# ab58626

MBP (Monoclonal Antibody). WB: 1:10,000	NEB	cat# e8032s
GFP. WB: 1:1,000	Abcam	cat# ab290
LAMP1 (H4A3). IF: 1:500	Santa Cruz Biotechnology	cat# sc-20011
LC3. IF: 1:500	MBL/Nordic Biosite	cat# PM036
CD63 (MX-49.129.5). WB: 1,000	Santa Cruz Biotechnology	cat# sc-5275
CD63 (H5C6). IF: 1:200	DSHB	cat# H5C6-s
GM130 (P-20). WB: 1:1,000	Santa Cruz Biotechnology	cat# sc-16268
CD9 (C-4). WB: 1:1,000	Santa Cruz Biotechnology	cat# sc-13118
HS1BP3. IHC: 1:200.	Novus Biologicals	cat# NB100-2415
Anti-Rabbit IgG (Starbright Blue 700). WB: 1:5,000	Bio-Rad	cat# 12004161
IgG (H+L) Cross-Adsorbed Donkey anti-Mouse, DyLight™ 800, Invitrogen™. WB: 1:5,000	Invitrogen	cat# SA5-10172
Donkey anti-Rabbit IgG (H+L) Cross-Adsorbed Secondary Antibody, DyLight™ 680. WB: 1:5,000	Invitrogen	cat# SA5-10042
Peroxidase AffiniPure Goat Anti-Mouse IgG (H+L) (HRP): WB: 1:5,000	Jackson ImmunoResearch	cat# 115-035-003
Goat anti-Mouse IgG (H+L) Highly Cross-Adsorbed Secondary Antibody, Alexa Fluor™ 546. IF: 1:500	Invitrogen	cat# A-11030
Donkey anti-Rabbit IgG (H+L) Highly Cross-Adsorbed Secondary Antibody, Alexa Fluor™ 488. IF: 1:500	Invitrogen	cat# A-21206
Goat anti-Rabbit IgG (H+L) Highly Cross-Adsorbed Secondary Antibody, Alexa Fluor™ 488. IF: 1:500	Invitrogen	cat# A-11034
Donkey anti-Mouse IgG (H+L) Highly Cross-Adsorbed Secondary Antibody, Alexa Fluor™ 647. IF: 1:500	Invitrogen	cat# A-31571
**Oligonucleotides and other sequence-based reagents**		
PCR primers	This study	Table EV1
qPCR primer: TKS5 (SH3PXD2A)	Qiagen	cat# QT00029764
qPCR primer: Cortactin (CCTN)	Qiagen	cat# QT00045766
qPCR primer: SDHA	Qiagen	cat# QT00059486
qPCR primer: HS1BP3	Qiagen	cat# QT00094899
siCTRL (5’UGGUUUACAUGUCGACUAA3’)	Dharmacon	cat# D-001810-01-20
siHS1BP3 (5’UGAAGAGGCUUUCGACUUU3’)	Dharmacon	cat# J-013029-10-0020
**Chemicals, Enzymes and other reagents**		
Alexa Fluor™ 633 Phalloidin.IF: 1:200,	Invitrogen	cat# A22284
Hoechst 33342. 0.1 ng/ml	Sigma Aldrich	cat# 23491-52-3
Bafilomycin A1. 100 nM	AH Diagnostics	cat# BML-CM110
Ham's F-12K (Kaighn's) Medium	Gibco,	cat# 21127030
RPMI 1640 Medium	Gibco	cat# 61870-044
Fetal Bovine Serum. 10%	Sigma Aldrich	cat# F7524
Penicillin-Streptomycin mix. 1%	Thermo- Fisher Scientific	cat# 15140122
Earle's Balanced Salt Solution (EBSS)	Gibco	cat# 24010043
Lipofectamine 2000	Invitrogen	cat# 11668019
Puromycin. 2 µg/ml	Sigma-Aldrich	cat# P7255-25MG
Gibson Assembly master mix	NEB	cat# E2611L
BbsI	NEB	cat# R0539S
Gateway™ LR Clonase™ reaction	Invitrogen	cat# 11791-100
QuikChange II site-directed mutagenesis kit	Agilent	cat# 210513

Xtreme-GENE DNA 9 Transfection Reagent	Roche	cat# XTG9-RO
Geneticin. 0.5 mg/ml	Fisher Scientific AS	cat# 11-811-031
Zeocin. 100 µg/ml	ThermoFisher Scientific	cat# R25001
Tris-HCl	Sigma-Aldrich	cat# T1378-1KG
NaCl	Sigma-Aldrich	cat# 71376
EDTA	Sigma-Aldrich	cat# 60-00-4
Nonidet P-40 Substitute	Roche	cat# 11754599001
Triton X-100	Sigma-Aldrich	cat# 9002-93-1
ChromoTek GFP-Trap® Agarose beads	Proteintech	cat# Gta-20
complete EDTA free protease inhibitor cocktail	Roche	cat# 5056489001
Glutathione Sepharose® 4B beads	Sigma Aldrich	cat# GE17-0756-01
amylose-resin beads	NEB	cat# E8021L
Poly-Prep® Chromatography Columns	Bio-Rad	cat# 7311555
Laemmli Sample Buffer	Bio-Rad	cat# 1610747
DTT	(Roche	cat# 10708984001
Pierce™ BCA™ Protein assay kit	VWR	cat# 786-0000
Allprotect tissue reagent	Qiagen	cat# 76.405
RNeasy plus mini kit	Qiagen	cat# 74136
AllPrep DNA/RNA/Protein Mini Kit	Qiagen	cat# 80004
RNase-Free DNase Set	Qiagen	cat# 79254
SuperScript™ III Reverse Transcriptase	Thermo fisher scientific	cat# 18080085
KAPA SYBR® FAST qPCR Kit	KAPA BIOSYSTEMS	cat# KK4601
4-20% gradient acrylamide gel	BioRad	cat# 5678094
non-fat milk	VWR	cat# A0830.0500
casein	Sigma Aldrich	cat# C7078-500G
% bovine serum albumin	Sigma Aldrich	cat# A1470-100G
West Pico PLUS Chemiluminescent Substrate	Thermo Fisher	cat# 34577
ProLong™ Diamond Antifade Mountant	Invitrogen	cat# P36961
NaAzide	VWR	cat# AA14314-36
poly-d-lysine	Merck	cat# P6403
Thiazolyl Blue Tetrazolium Bromide,	Merck	cat# M2128
Gelatin Oregon Green 488-conjugate	Invitrogen	cat# G-13186
AmiconUltra-15 centrifugal filter unit	Merck	cat# UFC 90102410 K cutoff
Mitomycin C	Calbiochem	cat# U75820
**Software**		
Fiji	[22]	
CellProfiler (v4.2 and 4.2.5)	[23]	
Image Studio™Lite	LI-COR	
Pymol version 3.1.3	Schrödinger, LLC	
Graphpad Prism (v10)	Dotmatics	
IncuCyte® Software S3	Sartorius	
QuPath (v0.5.0)	[24]	
**Other**		
TissueRuptor I	Qiagen	
ChemiDoc MP (BioRad) imaging system	Bio-Rad	
Odyssey ® CLx	LI-COR®	
Coverslips 12 mm diameter	VWR	cat# MENZCB00120RA020

ImageLock™ plates	Essen Bioscience	cat# 4379
Incucyte® 96-well WoundMaker tool	Essen Bioscience	
IncuCyte® S3 Live Cell microscope	Sartorius	
Nikon Ti2-E microscope with a Yokogawa CSU-W1 SoRa spinning disk	Nikon	
Nikon CREST X-Light V3 spinning disk	Nikon	
CFx96 real-time PCR system	Bio-Rad	
Invitrogen™ Countess™ Cell Counting Chamber Slides	Invitrogen	cat# 10399053
Dako Antibody Diluent	Agilent	cat# S0809
Dako PT-link system with Lab Vision™ PT Module	Agilent	
EnVision™ FLEX Target Retrieval Solution	Agilent	cat# K8004

Figure S1

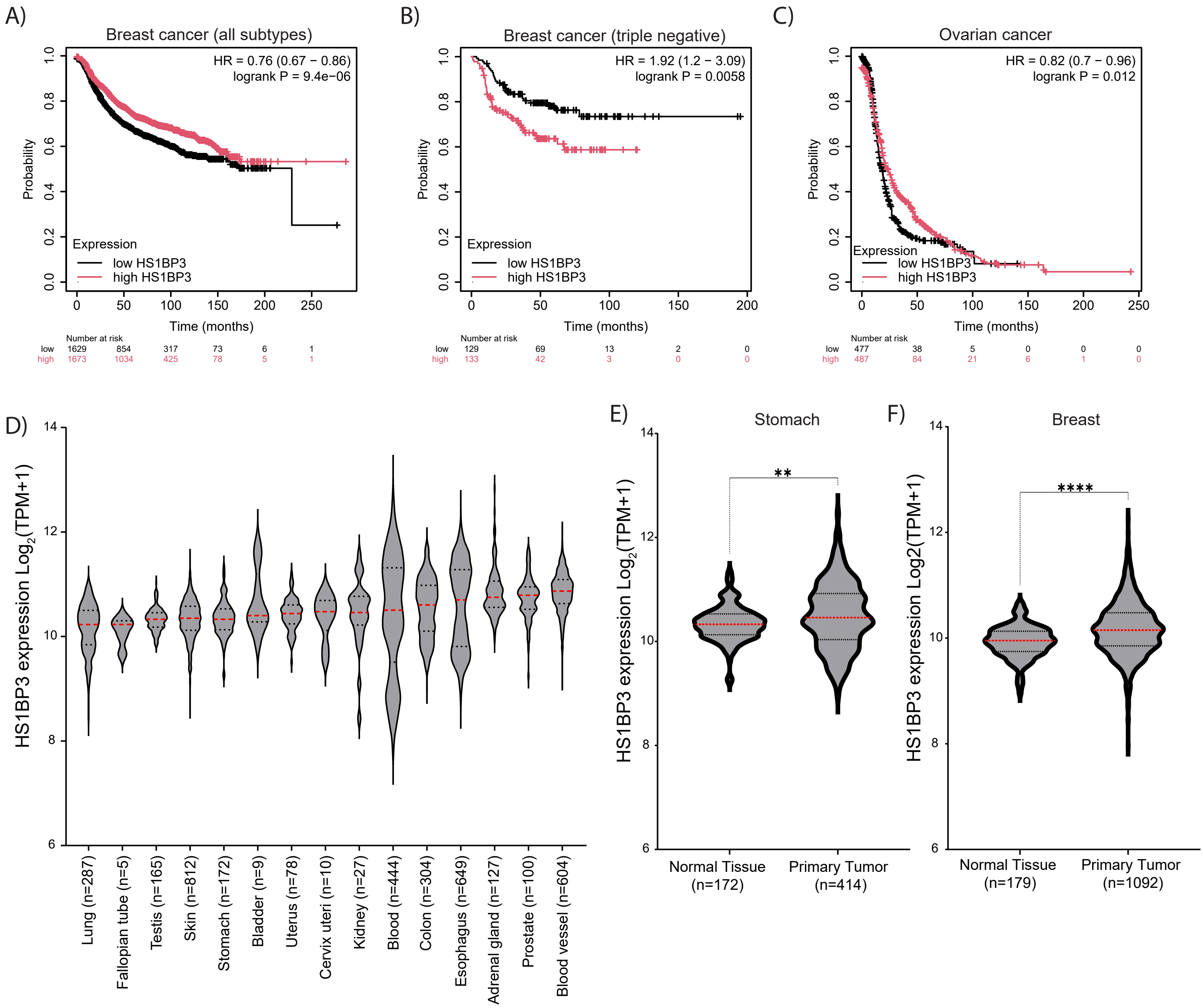
A)
Breast cancer (all subtypes)
HR = 0.76 (0.67 − 0.86)
logrank P = 9.4e−06
Probability
Expression
low HS1BP3
high HS1BP3
Time (months)
Number at risk
low 1629 854 317 73 6 1
high 1673 1034 425 78 5 1
B)
Breast cancer (triple negative)
HR = 1.92 (1.2 − 3.09)
logrank P = 0.0058
Probability
Expression
low HS1BP3
high HS1BP3
Time (months)
Number at risk
low 129 69 13 2 0
high 133 42 3 0 0
C)
Ovarian cancer
HR = 0.82 (0.7 − 0.96)
logrank P = 0.012
Probability
Expression
low HS1BP3
high HS1BP3
Time (months)
Number at risk
low 477 38 5 0 0 0
high 487 84 21 6 1 0
D)
HS1BP3 expression Log2(TPM+1)
Lung (n=287)
Fallopian tube (n=5)
Testis (n=165)
Skin (n=812)
Stomach (n=172)
Bladder (n=9)
Uterus (n=78)
Cervix uteri (n=10)
Kidney (n=27)
Blood (n=444)
Colon (n=304)
Esophagus (n=649)
Adrenal gland (n=127)
Prostate (n=100)
Blood vessel (n=604)
E)
Stomach
HS1BP3 expression Log2(TPM+1)
**
Normal Tissue (n=172)
Primary Tumor (n=414)
F)
Breast
HS1BP3 expression Log2(TPM+1)
****
Normal Tissue (n=179)
Primary Tumor (n=1092)

Figure S1: *HS1BP3 levels is predictive for patient outcomes in breast and ovarian cancer.* (**A-C**) Kaplan Meier plots showing the probability of survival in patients with breast cancer (all subtypes) (A), triple negative breast (B) and ovarian cancer (C) according to high (T3=third highest expressing tertile) and low (T1= third lowest expressing tertile) mRNA expression of HS1BP3 in tumour at time of detection. HR=Hazard ratio and p-value were determined with Cox proportional hazards regression. Plots are made in the KM plotter database. (**D**) Graph displaying the median and interquartile range of bulk tissue gene expression (TPM= transcripts per million + 1) of HS1BP3 across the top 15 most-HS1BP3 expressing human tissues according to median levels. The data is extracted from GTex portal. (**E-F**) Graph displaying the median and interquartile range of bulk tissue gene expression of HS1BP3 in healthy (GTex portal database) and cancerous (TCGA database) tissues of gastric (E) and breast (F). (E,F) p-values were determined using unpaired t-test. **= P <0.01; ****= P<0.0001.

Figure S2

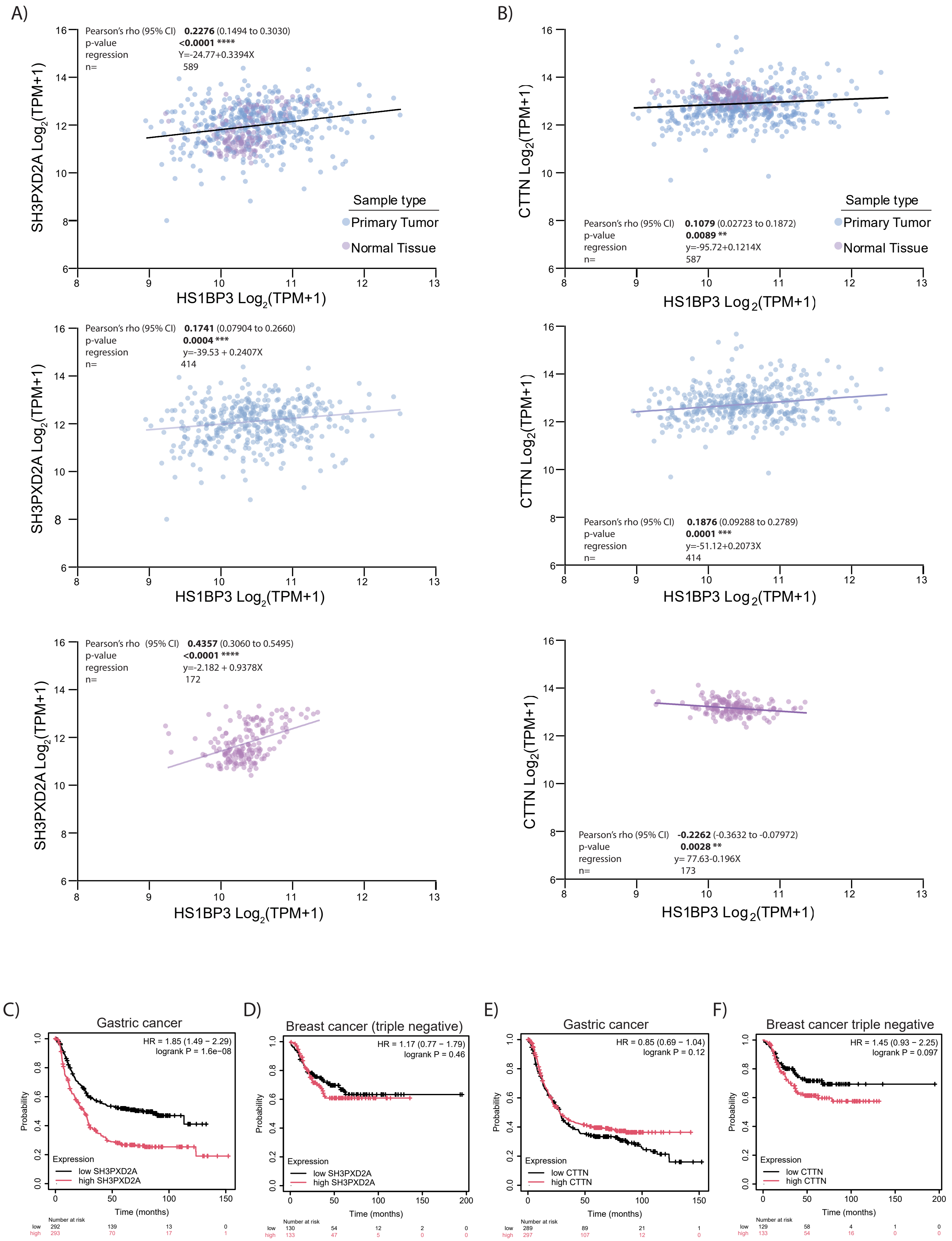

A)
Pearson's rho (95% CI) 0.2276 (0.1494 to 0.3030)
p-value <0.0001 ****
regression Y=-24.77+0.3394X
n= 589
SH3PXD2A Log2(TPM+1)
HS1BP3 Log2(TPM+1)
Sample type
Primary Tumor
Normal Tissue
B)
CTTN Log2(TPM+1)
HS1BP3 Log2(TPM+1)
Pearson's rho (95% CI) 0.1079 (0.02723 to 0.1872)
p-value 0.0089 **
regression y=-95.72+0.1214X
n= 587
Sample type
Primary Tumor
Normal Tissue
Pearson's rho (95% CI) 0.1741 (0.07904 to 0.2660)
p-value 0.0004 ***
regression y=-39.53 + 0.2407X
n= 414
SH3PXD2A Log2(TPM+1)
HS1BP3 Log2(TPM+1)
CTTN Log2(TPM+1)
Pearson's rho (95% CI) 0.1876 (0.09288 to 0.2789)
p-value 0.0001 ***
regression y=-51.12+0.2073X
n= 414
HS1BP3 Log2(TPM+1)
Pearson's rho (95% CI) 0.4357 (0.3060 to 0.5495)
p-value <0.0001 ****
regression y=-2.182 + 0.9378X
n= 172
SH3PXD2A Log2(TPM+1)
HS1BP3 Log2(TPM+1)
CTTN Log2(TPM+1)
Pearson's rho (95% CI) -0.2262 (-0.3632 to -0.07972)
p-value 0.0028 **
regression y= 77.63-0.196X
n= 173
HS1BP3 Log2(TPM+1)
C)
Gastric cancer
HR = 1.85 (1.49 − 2.29)
logrank P = 1.6e−08
Probability
Expression
low SH3PXD2A
high SH3PXD2A
Time (months)
Number at risk
low 292 139 13 0
high 293 70 17 1
D)
Breast cancer (triple negative)
HR = 1.17 (0.77 − 1.79)
logrank P = 0.46
Probability
Expression
low SH3PXD2A
high SH3PXD2A
Time (months)
Number at risk
low 130 54 12 2 0
high 133 47 5 0 0
E)
Gastric cancer
HR = 0.85 (0.69 − 1.04)
logrank P = 0.12
Probability
Expression
low CTTN
high CTTN
Time (months)
Number at risk
low 289 89 21 1
high 297 107 12 0
F)
Breast cancer triple negative
HR = 1.45 (0.93 − 2.25)
logrank P = 0.097
Probability
Expression
low CTTN
high CTTN
Time (months)
Number at risk
low 129 58 4 1 0
high 133 54 16 0 0

Figure S2: *HS1BP3 and SH3PXD2A (TKS5) gene expressions correlate in gastric tissues*. (**A-B**) Regression plots showing the Log_2 gene transcripts per million+1 (TPM+1) of HS1BP3 correlated to the Log_2 gene transcripts per million+1 (TPM+1) of SH3PXD2A (A) and CTTN (cortactin; B) in gastric tissues. The data is derived from TCGA (Gastric adenocarcinoma) and GTEx portal (healthy gastric tissue). A two-tailed Pearson's correlation rho coefficient with 95% CI (confidence interval) and p-values are listed together with a simple linear regression line of best fit. (**C-F)** Kaplan Meier plots showing the probability of survival in patients with gastric (C; E) and triple negative breast cancer (D; F) according to high (T3=third highest expressing tertile) and low (T1= third lowest expressing tertile) mRNA expression of *SH3PXD2A* (C-D) or *CTTN* (E-F) in tumour at time of detection. HR=Hazard ratio and p-value were determined with Cox proportional hazards regression. Plots are made in the KM plotter database. **= P <0.01; ***= P <0.001; ****= P<0.0001.

Figure S3

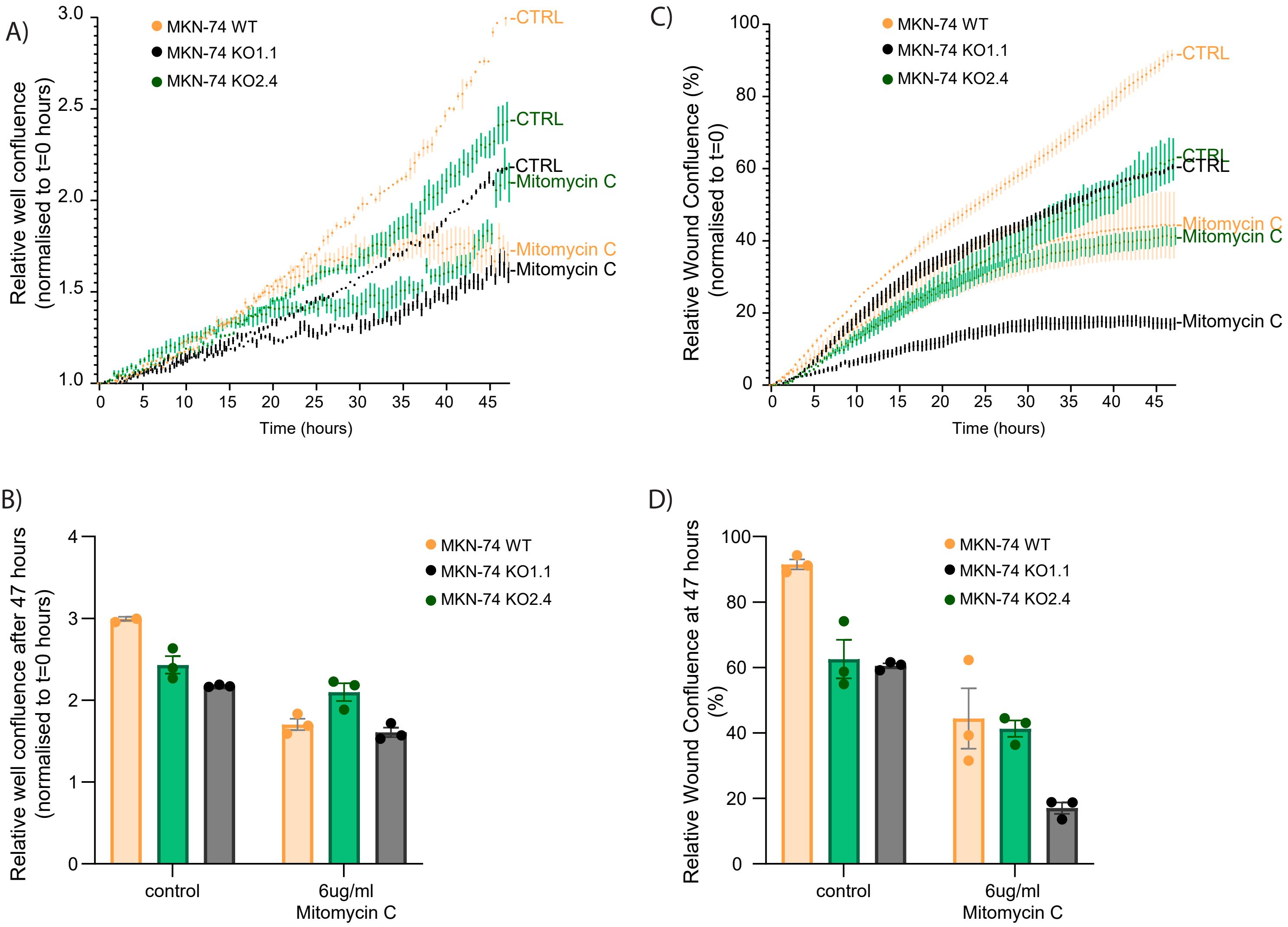

A)
MKN-74 WT
MKN-74 KO1.1
MKN-74 KO2.4
CTRL
CTRL
CTRL
Mitomycin C
Mitomycin C
Mitomycin C
Relative well confluence
(normalised to t=0 hours)
Time (hours)
C)
MKN-74 WT
MKN-74 KO1.1
MKN-74 KO2.4
CTRL
CTRL
CTRL
Mitomycin C
Mitomycin C
Mitomycin C
Relative Wound Confluence (%)
(normalised to t=0)
Time (hours)
B)
MKN-74 WT
MKN-74 KO1.1
MKN-74 KO2.4
Relative well confluence after 47 hours
(normalised to t=0 hours)
control
6ug/ml
Mitomycin C
D)
MKN-74 WT
MKN-74 KO1.1
MKN-74 KO2.4
Relative Wound Confluence at 47 hours
(%)
control
6ug/ml
Mitomycin C

Figure S3: *HS1BP3 regulates the proliferation of MKN-74 cells*. *(***A-B**) MKN-74 WT and HS1BP3 KO clones (1.1 and 2.4) were seeded sparsely in 96-well plates for live cell microscopy to observe their proliferation rate as mean relative confluence every 30 minutes over 47 hours. Plots show the relative well confluency up to 47 hours (A) and final well confluence at 47 hours (B). n=1 (2-3 technical replicates as indicated by the number of dots in B). (**C-D)** MKN-74 WT and HS1BP3 KO clones (1.1 and 2.4) were seeded confluently in 96-well plates and left overnight before creating wounds and imaged by live cell microscopy every 30 minutes over 47 hours to quantify the filling of the wound. Plots show the relative wound confluence of each well up to 47 hours after wound scratch (C) and the final relative wound confluence at 47 hours (D). n=1 (3 technical replicates). (A-D) data show mean ± standard error of the mean with individual data points corresponding to a single well (technical replicate).

Figure S4

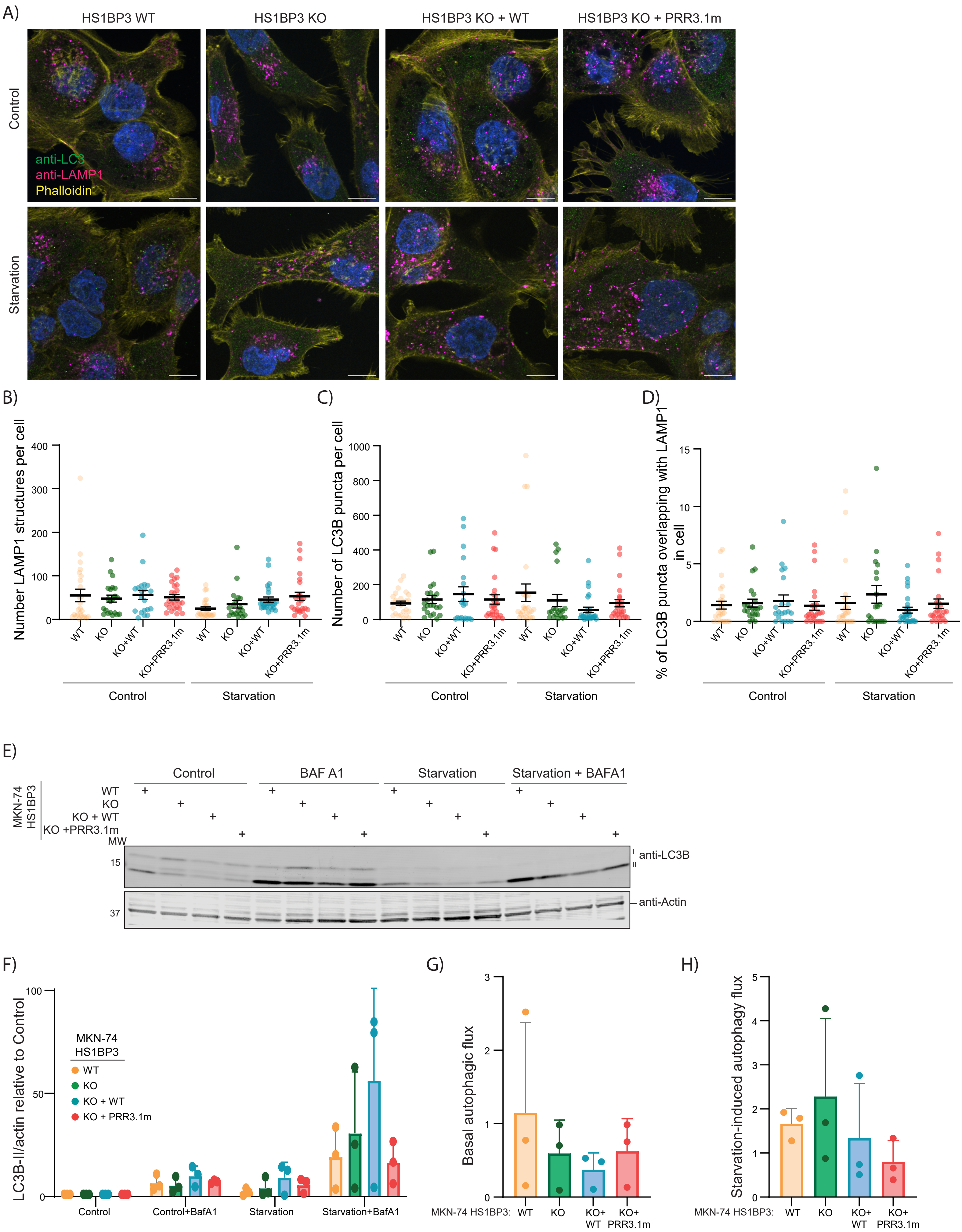

A)
HS1BP3 WT
HS1BP3 KO
HS1BP3 KO + WT
HS1BP3 KO + PRR3.1m
Control
Starvation
anti-LC3
anti-LAMP1
Phalloidin
B)
Number LAMP1 structures per cell
0
100
200
300
400
WT
KO
KO+WT
KO+PRR3.1m
Control
Starvation
C)
Number of LC3B puncta per cell
0
200
400
600
800
1000
WT
KO
KO+WT
KO+PRR3.1m
Control
Starvation
D)
% of LC3B puncta overlapping with LAMP1 in cell
0
5
10
15
WT
KO
KO+WT
KO+PRR3.1m
Control
Starvation
E)
Control
BAF A1
Starvation
Starvation + BAFA1
MKN-74 HS1BP3
WT
KO
KO + WT
KO +PRR3.1m
MW
15
37
I
II
anti-LC3B
anti-Actin
F)
LC3B-II/actin relative to Control
0
50
100
MKN-74 HS1BP3
WT
KO
KO + WT
KO + PRR3.1m
Control
Control+BafA1
Starvation
Starvation+BafA1
G)
Basal autophagic flux
0
1
2
3
MKN-74 HS1BP3:
WT
KO
KO+ WT
KO+ PRR3.1m
H)
Starvation-induced autophagy flux
0
1
2
3
4
5
MKN-74 HS1BP3:
WT
KO
KO+ WT
KO+ PRR3.1m

Figure S4: *HS1BP3 KO or expression of the PRR3.1-mutant has no effect on autophagy in gastric adenocarcinoma*. (**A**) Representative images of MKN-74 cells with HS1BP3 WT and KO 2.4 with HS1BP3 WT and PRR3.1 mutant. The cells were treated with EBSS for two hours for starvation, followed by immunofluorescence with antibodies anti-LC3B and anti-LAMP1 and staining with Phalloidin-633 and Hoechst, followed by acquisition with a Nikon Ti2-E microscope with a Yokogawa CSU-W1 SoRa spinning disk 100×/1.45 NA oil immersion objective. Scale bar: 10 µm. (**B**) Quantification of the number of LAMP1 structures per cell. (**C**) Quantification of the number of LC3B puncta per cell. (**D**) Quantification of the percentage of LC3B structures colocalising with LAMP1 structures. Data are mean ± standard error of the mean with individual data points corresponding to a single field of view (n=3, 29-357 cells were quantified in each condition). (**E**) Representative immunoblots of the indicated antibodies in MKN-74 WT, HS1BP3 KO 2.4, and HS1BP3 KO 2.4 rescue with HS1BP3 WT and PRR3.1-mutant cell line treated with 2 hours starvation with and without 100 nM Bafilomycin A1 (BAFA1) to demonstrate the autophagy flux of the indicated cells, n=3. (**F**) quantification of (E) showing level of LC3B-II normalised to actin levels and relative to control condition of the indicated cell line. (**G**) Quantified basal autophagy flux from (E) measured as the level of LC3B-II/actin in Control+BafA1 condition subtracted with the level of LC3B-II/actin in control condition per cell line. (**H**) Quantified starvation-induced autophagy flux from (E) measured as level of LC3B-II/actin in Starvation+BafA1 condition subtracted with level of LC3B-II/actin in Starvation condition per cell line. (F-H) Data are mean ± Standard deviation of the mean with individual data points corresponding to each replicate. The statistical significance was calculated with ordinary one-way ANOVA followed by Tukey's multiple comparison test (B-D), two-way ANOVA with Dunnett's multiple comparison test (F), or one-way ANOVA with Dunnett's multiple comparison test (G-H). Differences between the conditions were not significant (B-D, F-H).

Figure S5

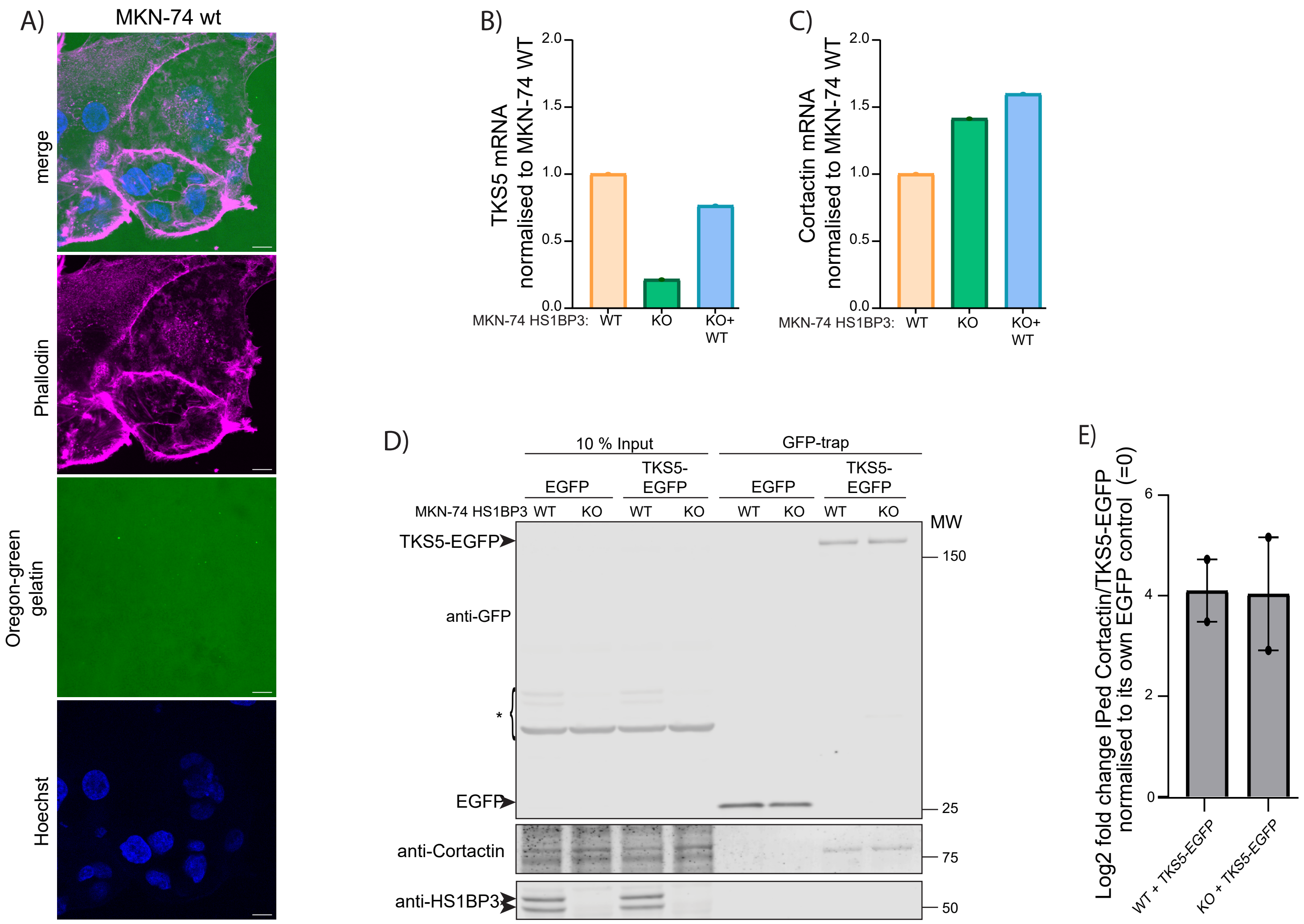

A)
MKN-74 wt
merge
Phallodin
Oregon-green gelatin
Hoechst
B)
TKS5 mRNA normalised to MKN-74 WT
2.0
1.5
1.0
0.5
0.0
MKN-74 HS1BP3: WT KO KO+ WT
C)
Cortactin mRNA normalised to MKN-74 WT
2.0
1.5
1.0
0.5
0.0
MKN-74 HS1BP3: WT KO KO+ WT
D)
10 % Input
GFP-trap
EGFP
TKS5-EGFP
MKN-74 HS1BP3 WT KO WT KO WT KO WT KO
MW
TKS5-EGFP
150
anti-GFP
*
EGFP
25
anti-Cortactin
75
anti-HS1BP3
50
E)
Log2 fold change IPed Cortactin/TKS5-EGFP normalised to its own EGFP control (=0)
6
4
2
0
WT + TKS5-EGFP
KO + TKS5-EGFP

Figure S5: *HS1BP3 levels do not affect cortactin-TKS5 protein interaction*. (**A**) representative immunofluorescence image of MKN-74 seeded on coverslips coated with Oregon-Green labelled gelatin for 17 hours before PFA-fixation and staining with Hoechst and phalloidin before visualising the cells by confocal imaging with a Yokogawa CSU-W1 SoRa spinning disk 60xWI water immersion objective. Scale bar is 10 µm. (**B-C**) The mRNA expression of *SH3PXD2A* (TKS5) (B) and *CTTN* (cortactin) (C) was analysed by quantitative PCR. n=1. (**D**) Lysates from MKN-74 WT and HS1BP3 KO (2.4) cells transiently transfected with pEGFP-N1 and pEGFP-TKS5-N1 were subjected to GFP-trap and immunoblot analysis of the indicated proteins. *=unspecific bands (**E**) quantification of (D) showing Log_2 fold difference in mean level of co-immunoprecipitated cortactin compared to level of immunoprecipitated TKS5-EGFP. The data is normalised to level of co-immunoprecipitated cortactin compared to level of immunoprecipitated EGFP in each cell type (=0, not shown). The data is mean ± standard deviation, n=2. Differences between the conditions were not significant and determined by paired t-test (E).

Figure S6

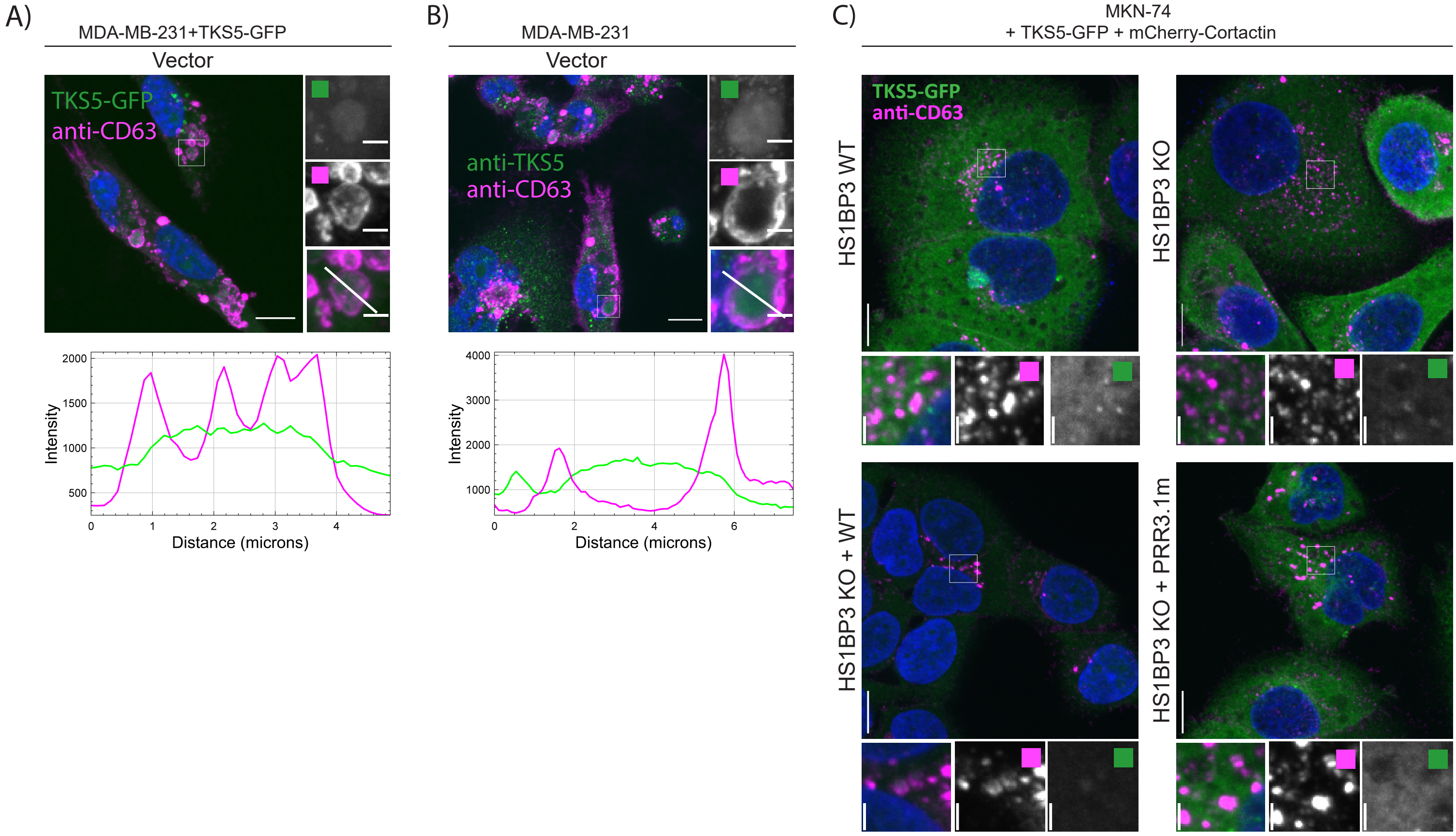

A)
MDA-MB-231+TKS5-GFP
Vector
TKS5-GFP
anti-CD63
Intensity
Distance (microns)
B)
MDA-MB-231
Vector
anti-TKS5
anti-CD63
Intensity
Distance (microns)
C)
MKN-74
+ TKS5-GFP + mCherry-Cortactin
TKS5-GFP
anti-CD63
HS1BP3 WT
HS1BP3 KO
HS1BP3 KO + WT
HS1BP3 KO + PRR3.1m

Figure S6: *TKS5 localise to CD63-positive structures*. (**A-B**) Representative immunofluorescence images of MDA-MB-231 overexpressing a negative control vector with (A) or without (B) stable expression of TKS5-GFP. The cells were PFA-fixed followed by antibody staining with anti-CD63 with (B) or without anti-TKS5 staining. Pixel intensity plots for line in the inset. (**C**) Representative immunofluorescence images of MKN-74 WT stably overexpressing TKS5-GFP and mCherry-Cortactin. Cells were PFA-fixed followed by antibody staining with anti-CD63 prior to imaging. (A-C) Images are taken with a Nikon CREST X-Light V3 spinning disk microscope using a 60× oil objective (NA 1.42). Maximum intensity projection is shown. Scale bar is 10 μm in the main figure and 2 μm in the inset.
